# Stability-Mediated Epistasis Restricts Accessible Mutational Pathways in the Functional Evolution of Avian Hemoglobin

**DOI:** 10.1093/molbev/msx085

**Published:** 2017-02-13

**Authors:** Amit Kumar, Chandrasekhar Natarajan, Hideaki Moriyama, Christopher C. Witt, Roy E. Weber, Angela Fago, Jay F. Storz

**Affiliations:** 1 School of Biological Sciences, University of Nebraska, Lincoln, NE; 2 Department of Biology, University of New Mexico, Albuquerque, NM; 3 Museum of Southwestern Biology, University of New Mexico, Albuquerque, NM; 4 Zoophysiology, Department of Bioscience, Aarhus University, Aarhus, Denmark

**Keywords:** epistasis, hemoglobin, fitness landscape, pleiotropy, nightjars, Caprimulgidae

## Abstract

If the fitness effects of amino acid mutations are conditional on genetic background, then mutations can have different effects depending on the sequential order in which they occur during evolutionary transitions in protein function. A key question concerns the fraction of possible mutational pathways connecting alternative functional states that involve transient reductions in fitness. Here we examine the functional effects of multiple amino acid substitutions that contributed to an evolutionary transition in the oxygenation properties of avian hemoglobin (Hb). The set of causative changes included mutations at intradimer interfaces of the Hb tetramer. Replacements at such sites may be especially likely to have epistatic effects on Hb function since residues at intersubunit interfaces are enmeshed in networks of salt bridges and hydrogen bonds between like and unlike subunits; mutational reconfigurations of these atomic contacts can affect allosteric transitions in quaternary structure and the propensity for tetramer–dimer dissociation. We used ancestral protein resurrection in conjunction with a combinatorial protein engineering approach to synthesize genotypes representing the complete set of mutational intermediates in all possible forward pathways that connect functionally distinct ancestral and descendent genotypes. The experiments revealed that 1/2 of all possible forward pathways included mutational intermediates with aberrant functional properties because particular combinations of mutations promoted tetramer–dimer dissociation. The subset of mutational pathways with unstable intermediates may be selectively inaccessible, representing evolutionary roads not taken. The experimental results also demonstrate how epistasis for particular functional properties of proteins may be mediated indirectly by mutational effects on quaternary structural stability.

## Introduction

When an evolutionary transition in protein function involves multiple mutational steps, a number of important questions can be addressed by experimentally examining the full set of possible intermediate genotypes that connect the ancestral starting point and the evolved endpoint (Weinreich 2010; Weinreich, et al. 2013; de Visser and Krug 2014; Hartl 2014). For example, Are particular endpoints selectively accessible from many different ancestral starting points? What fraction of possible pathways connecting ancestral and descendant genotypes involve transient reductions in fitness? What fraction of possible pathways could never be realized—representing evolutionary “roads not taken”—because they include nonfunctional/inviable intermediate steps? (Maynard Smith 1970; DePristo et al. 2005; Poelwijk et al. 2007; Kondrashov and Kondrashov 2015). These are fundamentally questions about the form and prevalence of intramolecular epistasis (nonadditive interactions between mutant sites in the same protein) and its role in shaping evolutionary trajectories through protein sequence space (Weinreich et al. 2005; Wagner 2008; Bloom and Arnold 2009; Carneiro and Hartl 2010; Harms and Thornton 2013; Kondrashov and Kondrashov 2015; Miton and Tokuriki 2016; Starr and Thornton 2016; Storz 2016a).

If *n* mutations are associated with an evolved change in protein function, and if a binary combination of ancestral/derived amino acids are possible at each site, then the key question is whether all *n* mutations have either neutral or beneficial effects in each of the 2^*n*^ possible multi-allelic combinations. If the sign of a mutation’s fitness effect depends on the allelic state at one or more other sites [“sign epistasis” (Weinreich et al. 2005)], then a subset of the *n*! mutational pathways connecting the ancestral and descendant genotypes will include intermediate steps with reduced fitness even if the fitness of the descendant genotype is greater than or equal to that of the ancestor (Malcolm et al. 1990; DePristo et al. 2005; Lunzer et al. 2005; Weinreich et al. 2005; Bridgham et al. 2006; Weinreich et al. 2006; Poelwijk et al. 2007; Bridgham et al. 2009; Lozovsky et al. 2009; Brown et al. 2010; da Silva et al. 2010; Weinreich 2010; Carroll et al. 2011; Kvitek and Sherlock 2011; Salverda et al. 2011; Dickinson et al. 2013; Gong et al. 2013; Schenk et al. 2013; de Visser and Krug 2014; Harms and Thornton 2014; Kondrashov and Kondrashov 2015; Palmer et al. 2015; Tufts et al. 2015; Bank et al. 2016; Wu et al. 2016).

There may be considerable scope for intramolecular epistasis during evolutionary shifts in the function of allosterically regulated, multimeric proteins like hemoglobin (Hb) because many residues are enmeshed in networks of site–site interactions that undergo discrete reconfigurations during ligation-dependent transitions in quaternary structure. The Hb tetramer is composed of two identical, semi-rigid α_1_β_1_ and α_2_β_2_ dimers that undergo a relative rotation of 15° during the oxygenation-linked transition between the deoxy (low affinity, T) conformation and the oxy (high affinity, R) conformation (Baldwin and Chothia 1979; Perutz 1989). Mutual rotation of the α_1_β_1_ and α_2_β_2_ dimers involves no appreciable change in the intradimer contact surfaces but substantial changes in intersubunit interactions at the α_1_β_2_ and α_2_β_1_ contacts (Pettigrew et al. 1982). The conformational equilibrium between the T- and R-states is central to the allosteric function of Hb as an O_2_-transport molecule, as it governs the cooperativity of O_2_-binding and the regulation of Hb-O_2_ affinity by allosteric effectors (nonheme ligands such as Cl^−^ ions and organic phosphates). Allosteric effectors reduce Hb-O_2_ affinity by preferentially binding and stabilizing Hb in the deoxy T-state, thereby shifting the allosteric equilibrium in favor of this low-affinity conformation.

Here we report an experimental analysis of multiple amino acid substitutions that occurred during an evolutionary transition in Hb function in South American nightjars (nocturnal birds in the family Caprimulgidae). We focus on one particular species, Tschudi’s nightjar (*Hydropsalis decussata*), a strictly lowland bird that has evolved a reduced Hb-O_2_ affinity relative to its highland sister species, the band-winged nightjar (*Hydropsalis longirostris*), which occurs at elevations ranging from ∼2000 m to 4400 m above sea level in the Andes (Schulenberg et al. 2007; Benham et al. 2011). We chose to dissect the molecular basis of this particular transition in protein function because ancestral sequence reconstruction indicated that the evolved reduction in Hb-O_2_ affinity in the *H*. *decussata* lineage involved the independent or joint effects of four amino acid substitutions, two of which involve intradimeric (α_1_β_1_ and α_2_β_2_) interfaces. Amino acid replacements at intradimeric contacts can affect the allosteric transition in quaternary structure and the propensity for tetramer–dimer dissociation (Fronticelli et al. 1994; Tsuruga et al. 1998; Vasquez et al. 1999; Chang et al. 2002; Yasuda et al. 2002; Shikama and Matsuoka 2003; Bellelli et al. 2006; Natarajan et al. 2013). Such replacements may be especially likely to have epistatic effects on Hb function since residues at intersubunit interfaces are involved in networks of salt bridges and hydrogen bonds with multiple residues in like and unlike subunits.

By reconstructing and resurrecting the Hb of the *H. decussata*/*H. longirostris* ancestor, we deduced that *H. decussata* evolved a derived reduction in Hb-O_2_ affinity. We then used a combinatorial protein engineering approach to synthesize genotypes representing all possible mutational intermediates in each of 4! = 24 forward pathways connecting the high-affinity ancestor to the low-affinity quadruple-mutant genotype of *H. decussata*. For each possible pathway, we experimentally characterized the trajectory of change in Hb-O_2_ affinity and a number of other properties (rates of autoxidation and measures of structural stability) that potentially trade-off with Hb-O_2_ affinity. The goal was to assess how mutational pleiotropy and epistasis influence evolutionary trajectories through protein sequence space. Specifically, we assessed the fraction of mutational pathways connecting functionally distinct ancestral and descendant genotypes that include potentially deleterious intermediate steps.

## Results and Discussion

### Evolved Functional Differences between Native Hbs of the High- and Low-Altitude Species

We start by presenting data on the functional and structural properties of native Hbs from the lowland *H. decussata* and the highland *H. longirostris*. We then explain how these results motivated additional protein-engineering experiments using site-directed mutagenesis.

Experiments based on a combination of isoelectric focusing (IEF) and tandem mass spectroscopy (MS/MS) revealed that *H. decussata* and *H. longirostris* both express two structurally distinct Hb isoforms in adult red blood cells (supplementary fig. S1, Supplementary Material online), consistent with data for most birds (Grispo et al. 2012; Opazo et al. 2015). The major and minor Hb isoforms (HbA and HbD, respectively) share identical β-type subunits, but the α-type subunits of HbA are encoded by the α^*A*^-globin gene and those of HbD are encoded by the closely linked α^*D*^-globin gene (Hoffmann and Storz 2007; Grispo et al. 2012; Opazo et al. 2015). There was no significant difference in the relative abundance of the two isoforms in the red blood cells of the two nightjar species; the percentage of total adult Hb accounted for by the minor HbD isoform (mean ± 1 SD) was 18.4% ± 4.1 for *H. decussata* (*n *=* *5 individual specimens) and 21.0% ± 5.3 for *H. longirostris* (*n *=* *6).

After isolating and purifying HbA and HbD from multiple specimens of each species, we measured oxygenation properties of the purified Hb solutions under standardized experimental conditions (Imai 1982; Mairbäurl and Weber 2012). Genetically based differences in Hb-O_2_ affinity can stem from changes in intrinsic O_2_-affinity and/or changes in the responsiveness to allosteric effectors like Cl^−^ ions and organic phosphates. To reveal the functional mechanisms that are responsible for observed differences in Hb-O_2_ affinity, we measured O_2_ equilibria of purified Hbs under four standardized treatments: (*i*) in the absence of allosteric effectors (stripped), (*ii*) in the presence of Cl^−^ ions, (*iii*) in the presence of inositol hexaphosphate (IHP) (a chemical analog of the endogenously produced inositol pentaphosphate) and (*iv*) in the simultaneous presence of Cl^−^ and IHP. This latter treatment is most relevant to *in vivo* conditions in avian red blood cells. In each treatment we estimated *P*_50_ (the O_2_ partial pressure [*P*O_2_] at which Hb is half-saturated), and the Hill coefficient, *n*_50_, an index of subunit cooperativity in the Hb tetramer.

**Fig. 1:**
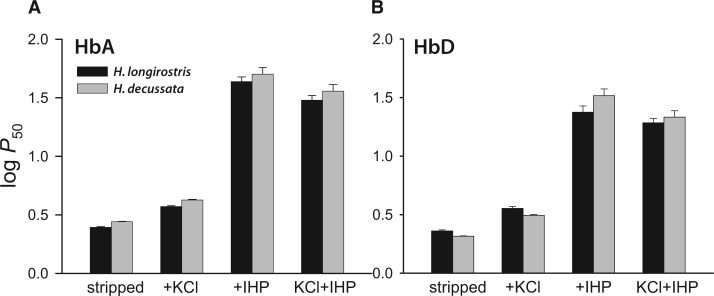
O_2_-affinities of HbA and HbD isoforms from high- and low-altitude nightjar species, *Hydropsalis longirostris* and *H. decussata*, respectively. (*A*) Log-transformed *P*_50_ values (± 1 SE) for purified HbA isoforms measured in 0.1 M HEPES buffer at pH 7.4, 37 °C, in the absence (stripped) and presence of allosteric effectors ([Cl^−^], 0.1 M; IHP/Hb tetramer ratio, 2.0; [heme], 0.3 mM). (*B*) *P*_50_ values for HbD isoforms (experimental conditions as in *A*).

The experiments revealed that HbD has a uniformly higher O_2_ affinity than HbA in both species (fig. 1 and table 1), a pattern of isoform differentiation that is consistent with data from other bird species (Grispo et al. 2012; Cheviron et al. 2014; Galen et al. 2015; Natarajan et al. 2015b; Opazo et al. 2015; Natarajan et al. 2016). Comparisons between the two nightjar species revealed no appreciable differences in the intrinsic O_2_ affinities of either HbA or HbD (as indicated by *P*_50_’s for the “stripped” Hbs); however, in the presence of IHP, O_2_-affinities of both isoforms were significantly higher (i.e., *P*_50_’s were lower) in the highland *H. longirostris* than in the lowland *H. decussata* (fig. 1 and table 1).

**Table 1:** O_2_ Affinities (*P*_50_, torr; mean ± SE) and Cooperativity Coefficients (*n*_50_) of Purified HbA and HbD Isoforms from High- and Low-Altitude Nightjar Species (*Hydropsalis longirostris* and *H. decussata*, respectively).

Species	Hb Isoform	Stripped		+ KCl		+ IHP		KCl + IHP		
		*P*_50_	*n*_50_	*P*_50_	*n*_50_	*P*_50_	*n*_50_	*P*_50_	*n*_50_
*H. longirostris*	HbA	2.48 ± 0.03	1.77 ± 0.04	3.73 ± 0.04	1.97 ± 0.05	43.55 ± 1.06	2.35 ± 0.11	30.21 ± 0.82	2.29 ± 0.14
	HbD	2.30 ± 0.05	1.67 ± 0.06	3.58 ± 0.10	1.81 ± 0.07	23.72 ± 0.92	2.32 ± 0.17	19.30 ± 0.55	2.34 ± 0.13
*H. decussata*	HbA	2.77 ± 0.02	1.87 ± 0.02	4.24 ± 0.04	2.12 ± 0.04	50.38 ± 1.64	2.01 ± 0.10	36.12 ± 1.30	2.13 ± 0.14
	HbD	2.07 ± 0.02	1.51 ± 0.03	3.13 ± 0.04	1.86 ± 0.04	32.88 ± 1.24	2.33 ± 0.17	21.54 ± 0.88	2.40 ± 0.24

Note.—O_2_ equilibria were measured in 0.1 mM HEPES buffer at pH 7.40, 37 °C, in the absence (stripped) and presence of Cl^−^ ions (0.1 M KCl]) and IHP (at 2-fold molar excess over tetrameric Hb). *P*_50_ and *n*_50_ values were derived from single O_2_ equilibrium curves, where each value was interpolated from linear Hill plots based on 4 or more equilibrium steps between 25 and 75% saturation.

### Structural Basis of the Evolved Change in Hb-O_2_ Affinity

**Fig. 2:**
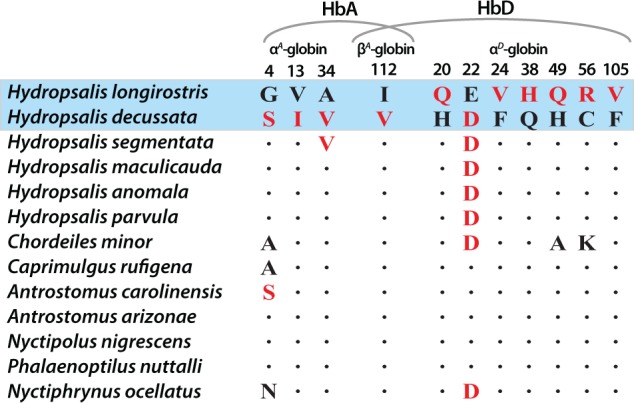
Amino acid substitutions that distinguish the HbA and HbD isoforms of *Hydropsalis longirostris* and *H. decussata* and amino acid states at orthologous sites in other New World nightjar species. At each site, derived (nonancestral) amino acids are denoted by red lettering. We used gene-specific phylogeny reconstructions to infer the polarity of character state changes at divergent sites in the α^*A*^-, α^*D*^-, and β^*A*^-globin genes (see supplementary fig. S2, Supplementary Material online).

By cloning and sequencing the full complement of adult-expressed globin genes in all nightjar specimens, we deduced that the observed difference in HbA O_2_ affinity between *H. longirostris* and *H. decussata* is attributable to the independent or combined effects of four amino acid substitutions (three in the α^*A*^-globin gene and one in β^*A*^-globin; fig. 2). Likewise, the observed difference in HbD O_2_ affinity between the two species is attributable to the independent or combined effects of eight substitutions (seven in α^*D*^-globin and one in β^*A*^-globin; fig. 2). Since HbA and HbD share the same β-chain subunit, the same β112Ile→Val (Iβ112V) substitution at site 112 distinguishes both isoforms of the two species. Given that the major HbA isoform accounts for ∼80% of total adult Hb in both species, we mainly focus on the functional evolution of HbA from this point forward.

To infer the polarity of character state changes at each of the sites that differ between the Hb isoforms of *H. decussata* and *H. longirostris*, we cloned and sequenced orthologs of α^*A*^-, α^*D*^-, and β^*A*^-globin from 11 other New World nightjar species, including four additional species in the genus *Hydropsalis* (fig. 2). Phylogenetic reconstructions of the globin sequences from the 13 nightjar species revealed a high level of genealogical discordance between the α^*A*^/α^*D*^ and β^*A*^-globin gene trees (supplementary fig. S2, Supplementary Material online). This pattern of genealogical discordance has been documented for other nuclear and mitochondrial genes in previous studies of nightjar systematics (Larsen et al. 2007; Han et al. 2010; Sigurdsson and Cracraft 2014) and is likely attributable to incomplete lineage sorting and/or introgressive hybridization.

In spite of the discordant tree topologies for the unlinked α- and β-type globin genes, the polarities of the four HbA amino acid changes were unambiguous (fig. 2 and supplementary fig. S2, Supplementary Material online). At each of the four sites that distinguish the HbA isoforms of *H. decussata* and *H. longirostris*, parsimony clearly suggests that *H. decussata* possesses the derived amino acid. Thus, even though *H. decussata* and *H. longirostris* conform to the well-documented adaptive trend where high-altitude bird species tend to have higher Hb-O_2_ affinities than their lowland relatives (Storz 2016b), this particular pair of nightjar species is unusual in that the low-altitude species evolved a derived reduction in Hb-O_2_ affinity and the high-altitude species retained the ancestral, high-affinity character state. In other studies of birds in which altitude-related changes in Hb function have been documented, the high-altitude species have evolved derived increases in Hb-O_2_ affinity (Projecto-Garcia et al. 2013; Galen et al. 2015; Natarajan et al. 2015b, 2016). There is no independent evidence to suggest that *H. decussata* descended from highland ancestors or that the evolved reduction in Hb-O_2_ affinity in this species constitutes an adaptation to low-altitude.

### Accessibility of Pathways through Sequence Space

Using the inferred character polarity for each of the four amino acid changes described above, we reconstructed and experimentally resurrected the HbA isoform of the *H. decussata*/*H. longirostris* ancestor. We then used a combinatorial protein engineering approach to synthesize genotypes representing all possible mutational intermediates in each of 24 forward pathways connecting the ancestral genotype “GVAI” to the quadruple-mutant genotype of *H. decussata*, “SIVV”. This required the synthesis, expression, and purification of 2^4 ^=^ ^16 recombinant Hb (rHb) mutants. We characterized the oxygenation properties for each of these 16 rHbs and we also measured several additional biochemical and biophysical properties that potentially trade-off with Hb-O_2_ affinity (autoxidation rates and indices of structural stability). The goal was to test whether changes in Hb-O_2_ affinity were consistently associated with changes in other properties and to characterize pleiotropic effects of affinity-altering mutations.

**Fig. 3:**
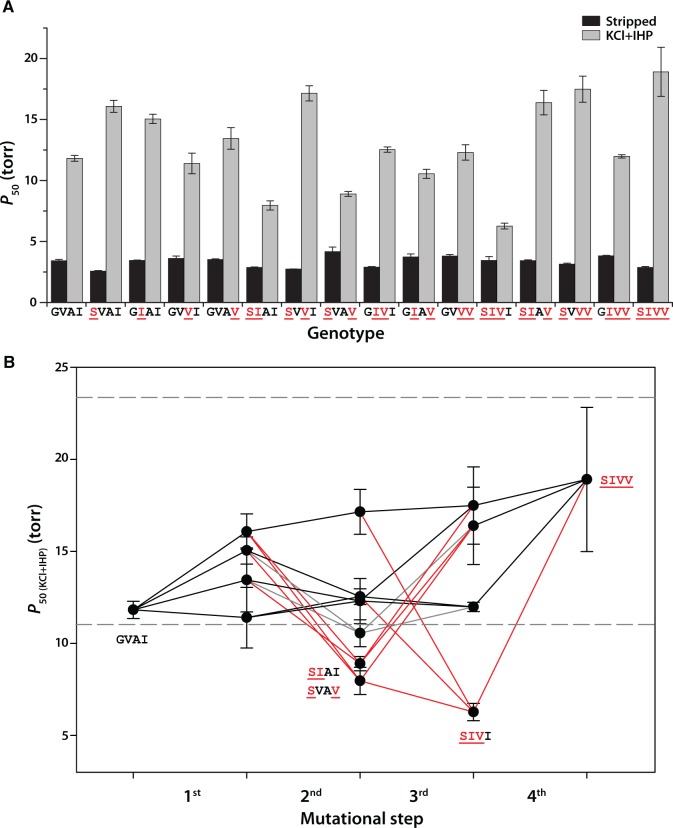
Sign epistasis for Hb-O_2_ affinity constrains the number of selectively accessible mutational pathways from the ancestral GVAI genotype to the descendent SIVV genotype. (*A*) O_2_-affinities (*P*_50_, torr; ± 1 SE) of 16 purified rHbs representing all possible genotypic combinations of bi-allelic variation at the four sites that distinguish GVAI and SIVV. Estimates of *P*_50_ for “stripped” Hbs provide measures of intrinsic O_2_ affinity, whereas estimates of *P*_50_ in the “KCl + IHP” treatment provide measures of Hb-O_2_ affinity in the presence of allosteric effectors. At each site, the derived (nonancestral) amino acids are underlined. (*B*) Trajectories of change in Hb-O_2_ affinity (indexed by *P*_50(KCl + IHP)_) in each of 24 forward pathways connecting the ancestral GVAI and the descendant SIVV. Dashed horizontal lines depict boundaries of the “neutral corridor” (see text for details). Of the 24 possible forward pathways, 12 included one or more mutational intermediates with values of *P*_50(KCl + IHP)_ that fell well below the floor of the neutral corridor (trajectories shown in red lines); these putatively inaccessible pathways included intermediates with highly aberrant functional properties (SIAI, SVAV, and SIVI). An additional four pathways passed through the GIAV genotype, which exhibited a marginally significant reduction in *P*_50(KCl + IHP)_ below the floor of the neutral corridor (trajectories shown in grey). Error bars denote 95% confidence intervals.

Consistent with the analysis of the native HbA isoforms, the quadruple-mutant SIVV genotype (identical to *H. decussata* HbA) exhibited a significantly lower O_2_-affinity than the ancestral GVAI genotype (identical to *H. longirostris* HbA) in the presence of allosteric effectors (*P*_50(KCl + IHP)_ was 1.6-fold higher; supplementary table S1, Supplementary Material online). Data for the full set of 16 rHbs revealed very little variation in intrinsic O_2_ affinity (indexed by *P*_50(stripped)_) but substantial variation in O_2_ affinity in the presence of allosteric effectors (indexed by *P*_50(KCl + IHP)_; fig. 3*A*); this variation was largely attributable to unusually low IHP sensitivities in a subset of double and triple mutants. In particular, two double-mutants (SIAI and SVAV) and one triple-mutant (SIVI) exhibited aberrant O_2_-binding properties, with *P*_50(KCl + IHP)_ values that were drastically lower than that of the ancestral GVAI genotype (supplementary table S1, Supplementary Material online). The *P*_50(KCl + IHP)_ values for these three genotypes are far below the range of naturally occurring values for avian HbA isoforms measured under identical conditions (Grispo et al. 2012; Natarajan et al. 2015b, 2016). The combination of an aberrantly high Hb-O_2_ affinity in the presence of IHP and low cooperativity (i.e., values of *n*_50_ close to 1.0) indicate that tissue O_2_ delivery would be impaired because Hb would remain highly saturated even at the low *P*O_2_ prevailing in systemic capillary beds. An increase in Hb-O_2_ affinity reduces the *P*O_2_ at which O_2_ is released, resulting in a diminished gradient for O_2_ diffusion from capillary blood to the tissue mitochondria. This is why human Hb mutants with unusually high O_2_-affinity are typically pathological (Wajcman and Galactéros 1996; Steinberg and Nagel 2009).

**Fig. 4:**
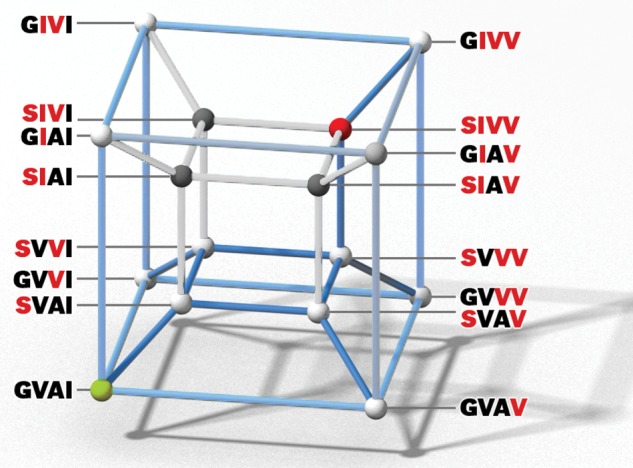
Four-dimensional depiction of sequence space, showing all possible mutational pathways connecting the high-affinity, ancestral GVAI genotype and the low-affinity, quadruple-mutant SIVV genotype (depicted by green and red nodes, respectively). All 16 genotypes are represented as nodes (vertices of the hypercube), with edges connecting genotypes that differ by a single point mutation. Putatively inaccessible pathways that pass through the SIAI, SVAV, and SIVI genotypes are denoted by light grey edges; pathways that pass through the GIAV genotype (which had a marginally significant reduction in *P*_50(KCl + IHP)_) are denoted by darker, bluish grey edges; accessible pathways, in which each successive mutational step resulted in an unchanged or reduced Hb-O_2_ affinity (increased *P*_50_) are denoted by blue edges.

Of the 24 possible forward pathways that connect the ancestral, high-affinity GVAI genotype and the derived, low-affinity SIVV genotype, a total of 12 pathways include the aberrant SIAI, SVAV, and/or SIVI genotypes as intermediate steps. Using the heuristic metaphor introduced by Malcolm et al. (1990), these pathways fall outside the so-called “neutral corridor”. For a given functional or structural property of a protein, the neutral corridor is defined by the range of values that fall within the interval between the lower (or upper) 95% confidence limit of the ancestral, start-point genotype and the upper (or lower) 95% confidence limit of the descendant, endpoint genotype (Malcolm et al. 1990). As shown in figure 3*B*, values of *P*_50(KCl + IHP)_ for the SIAI, SVAV, and SIVI genotypes fell well below the lower 95% confidence limit of the ancestral, high-affinity GVAI genotype, and an additional double-mutant genotype, GIAV, exhibited a marginally significant reduction in *P*_50(KCl + IHP)_ below this same threshold. These four genotypes exhibited lower IHP sensitivities than all the other 12 genotypes (supplementary table S1, Supplementary Material online), indicating reduced capacities for allosteric regulatory control. In the remaining forward pathways, each successive mutational step either results in a significant reduction in Hb-O_2_ affinity or a more-or-less unchanged Hb-O_2_ affinity (where observed differences were within the range of measurement error). The existence of these two distinct pathway types—those contained within the neutral corridor and those that include putatively deleterious intermediate steps—is attributable to the fact that the sign of some mutations’ phenotypic effects depend on which other substitutions have already occurred. All 24 pathways have the same ancestral starting point (GVAI), they all have the same endpoint (SIVV), and they all involve the same combination of four mutations; the different pathways are only distinguished by the sequential order in which the substitutions occurred. If the aberrant functional properties of the above-mentioned genotypes confer a reduced fitness, then sign-epistasis for Hb-O_2_ affinity would have the effect of restricting the selective accessibility of pathways leading from GVAI to SIVV, as depicted in a four-dimensional genotype space in fig. 4. These inferences about pathway accessibility are premised on two assumptions about evolutionary dynamics. First, the depictions in figures 3*B* and 4 assume that mutations are fixed sequentially on invariant genetic backgrounds, so mutational trajectories can be envisioned as a succession of discrete transitions between adjacent points in sequence space (Weinreich 2010). The second assumption is that once a mutation is fixed, it does not later revert to the ancestral state. The simultaneous fixation of two or more mutations and mutational reversions can potentially produce new connecting pathways in addition to the direct, forward pathways shown in figures 3*B* and 4 (Kimura 1985; Weinreich and Chao 2005; Weinreich et al. 2005; DePristo et al. 2007; Meer et al. 2010; Weinreich 2010; Franke et al. 2011; Covert et al. 2013; Palmer et al. 2015; McCandlish et al. 2016; Wu et al. 2016).

### Causes of Epistasis for Protein Function

**Fig. 5:**
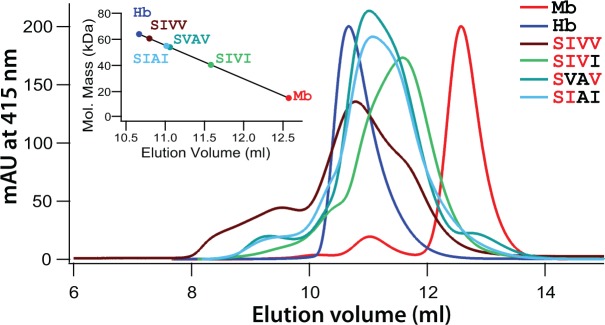
FPLC gel filtration elution profile for recombinant Hbs (rHbs) representing SIVV, the wild-type genotype of *H. decussata*, and three rHb mutants (SIAI, SVAV, and SIVI) that exhibited aberrant O_2_-binding properties. As discussed in the text, these three rHb mutants exhibited unusually high O_2_-affinities in the presence of allosteric effectors (i.e., low values of *P*_50(KCL + IHP)_), which suggested that they may have an increased propensity for tetramer–dimer dissociation. Monomeric horse myoglobin (Mb) and tetrameric human Hb were used as standard molecular weight markers. In the plot of elution peak against elution volume for the four rHbs, a shift in the equilibrium towards tetramer–dimer dissociation is indicated by elution volumes shifted towards the Mb value. The gel filtration elution profiles indicate that the three rHbs with suppressed sensitivities to IHP have an increased tendency to dissociate into dimers.

What is the biophysical basis of the observed epistasis for Hb-O_2_ affinity in the presence of allosteric effectors? IHP binds with 1:1 stoichiometry between the β-chain subunits of deoxyHb via charge-charge interactions with a set of highly conserved cationic residues (Arnone and Perutz 1974). Amino acid replacements that directly or indirectly inhibit phosphate binding will typically increase Hb-O_2_ affinity by shifting the allosteric equilibrium in favor of R-state oxyHb (Storz et al. 2009, 2010; Revsbech et al. 2013; Natarajan et al. 2013; Janecka et al. 2015; Natarajan et al. 2015a). However, none of the observed substitutions in *H. decussata* HbA directly affect IHP-binding sites in the central cavity. Instead, the combination of low cooperativity and reduced responsiveness to IHP (supplementary table S1, Supplementary Material online) suggest the hypothesis that the high-affinity rHb mutants are partly dissociating into α_1_β_1_ and α_2_β_2_ dimers. This is because oxygenation-linked transitions in quaternary structure provide the basis for both cooperative O_2_-binding and allosteric regulation via IHP-binding. If these allosteric properties are compromised, it suggests that the Hb samples may contain an equilibrium mixture of functionally intact Hb tetramers (whose O_2_-affinities are significantly reduced in the presence of IHP) and dissociated α_1_β_1_ and α_2_β_2_ dimers (whose O_2_-affinities are unaffected by the presence of IHP). To test this hypothesis, we performed a gel-filtration experiment to assess whether the rHb mutants with the three lowest *P*_50(KCl + IHP)_ values (SIAI, SVAV, and SIVI) exhibited higher rates of tetramer–dimer dissociation relative to the quadruple-mutant, SIVV (which had the highest *P*_50(KCl + IHP)_ value). Results of this experiment clearly demonstrated that the rHb mutants with low IHP sensitivity had elution volumes intermediate between values for tetrameric human Hb and monomeric myoglobin—which is indicative of tetramer–dimer dissociation—whereas SIVV had an elution volume similar to that of human Hb, the control for an intact, tetrameric assembly (fig. 5). These results indicate that the exceedingly low IHP sensitivities of SIAI, SVAV, and SIVI (and the sign epistasis for *P*_50(KCl + IHP)_) are not directly attributable to mutational effects on IHP binding. Instead, the unusually high O_2_-affinities in the presence of IHP are attributable to the indirect effects of tetramer–dimer dissociation. The Hb concentrations in our *in vitro* O_2_ equilibrium experiments are considerably lower than *in vivo* concentrations in circulating red blood cells, but if the same variation in quaternary structural stability is manifest *in vivo*, then our findings suggest that the subset of mutational pathways that include the unstable genotypes as intermediate steps may represent evolutionary dead ends. Even if the rate of tetramer–dimer dissociation is lower under *in vivo* conditions, our results demonstrate how—in principle—epistasis for particular functional properties of proteins may be mediated indirectly by mutational effects on stability of the tetrameric assembly.

### Insights into Structural Mechanisms

To decipher the causative effects of the four amino acid mutations, it is clear that we need to distinguish between their direct effects on Hb-O_2_ affinity and their indirect effects on Hb-O_2_ affinity that are mediated by effects on tetramer–dimer dissociation.

**Fig. 6:**
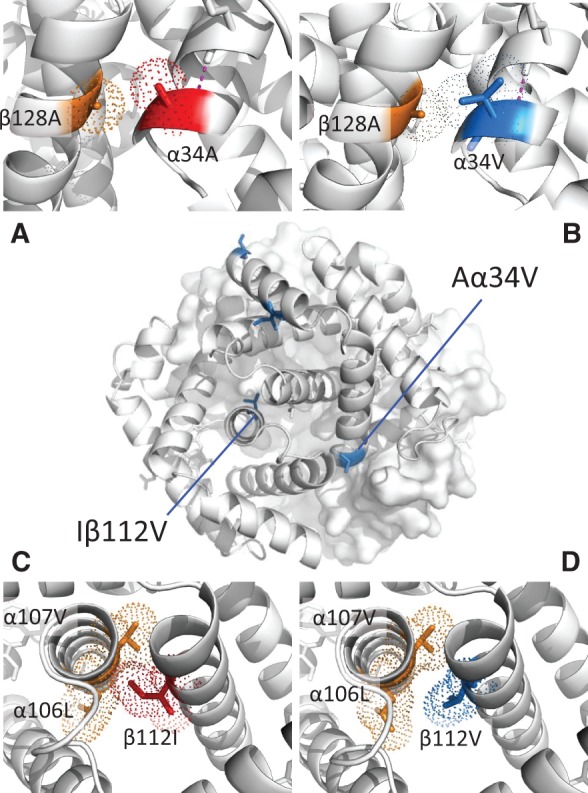
Amino acid mutations at intradimeric interfaces reduce Hb-O_2_ affinity via different structural mechanisms. (*A*,*B*) Replacing Ala with Val at α34V results in the gain of two additional intradimer atomic contacts per Hb tetramer, as Vα34 forms a van der Waals contact with Aβ128 in each αβ dimer. This stabilization of α_1_β_1_/α_2_β_2_ contacts is predicted to restrict allosteric motion, thereby reducing O_2_-affinity by increasing the free energy of the oxygenation-linked T→R transition in quaternary structure. (*C,D*) Replacing Ile with Val at β112 contributes to a destabilization of the R-state by eliminating two intradimeric van der Waals contacts (Iβ112::Vα107) per Hb tetramer.

Results of homology-based structural modeling suggest specific biophysical mechanisms by which each of the four amino acid substitutions contribute to the reduced O_2_ affinity of *H. decussata* HbA*.* The two substitutions in the α-chain A-helix (Gα4S and Vα13I) are predicted to reduce Hb-O_2_ affinity by eliminating specific atomic contacts in oxyHb, thereby reducing the relative stability of the R-state (supplementary fig. S3, Supplementary Material online). The other two amino acid substitutions, Aα34V and Iβ112V, affect intradimeric α_1_β_1_ and α_2_β_2_ contacts. The Aα34V substitution results in the gain of two additional intradimer atomic contacts per Hb tetramer in both the R- and T-states, as α34V forms a van der Waals contact with β128A in each of the constituent αβ dimers (fig. 6*A* and *B*). This stabilization of intradimeric contacts is predicted to restrict allosteric motion in the T→R transition in quaternary structure, thereby reducing O_2_-affinity by shifting the allosteric equilibrium in favor of the deoxy T-state. The Iβ112V substitution reduces Hb-O_2_ affinity by simultaneously destabilizing the R-state and stabilizing the T-state. The ancestral β112I forms an intradimeric van der Waals contact with α107V in oxy (R-state) Hb (fig. 6*C*), and it forms an intradimeric contact with α110A in deoxy (T-state) Hb. The Iβ112V substitution eliminates the intradimer contact with α107V in oxyHb (fig. 6*D*), which contributes to the destabilization of the R-state, and it produces an additional intradimeric contact between β112V and α106L in deoxyHb, which contributes to the stabilization of the T-state.

In addition to contributing to the reduced Hb-O_2_ affinity in *H. decussata*, the Iβ112V substitution also appears to affect the propensity for tetramer–dimer dissociation, although its effects are highly context dependent. On some backgrounds, the Iβ112V substitution reduced *P*_50(KCl + IHP)_ values below the floor of the neutral corridor (e.g., SVAI→SVAV and GIAI→GIAV); in other cases the same substitution produced equally dramatic, compensatory increases in *P*_50(KCl + IHP)_ (e.g., SIAI→SIAV and SIVI→SIVV). For example, the SIAI and SIVI genotypes exhibited the lowest *P*_50(KCl + IHP)_ values and the lowest sensitivities to IHP (supplementary table S1, Supplementary Material online); on these two backgrounds, the Iβ112V mutation increased *P*_50(KCl + IHP)_ 2.1-fold and 2.6-fold, respectively, and it increased IHP sensitivity 1.6-fold and 2.6-fold, respectively (supplementary table S1, Supplementary Material online). This dramatic rescue effect is likely not attributable to direct effects on IHP-binding; rather, the Iβ112V substitution increased responsiveness to IHP by inhibiting tetramer–dimer dissociation. In other words, restoration of allosteric regulatory capacity followed automatically from a restoration of the intact tetrameric assembly. These documented effects are consistent with other functional studies of β112 mutations in human Hb. For example, the Cβ112G mutation produces a 4-fold reduction in the tendency of human Hb to dissociate into dimers (Fronticelli et al. 1994). By contrast, Cβ112R and Cβ112F increase this tendency and are associated with hemolytic anemia (Adams et al. 1979; Brennan et al. 2002). Since tetramer–dimer dissociation involves the interdimeric α_1_β_2_/α_2_β_1_ interfaces, the data for human Hb mutants and the engineered nightjar Hb mutants indicate that perturbations of the intradimeric contacts are transmitted to contacts between dimers (Fronticelli et al. 1994; Vasquez et al. 1999; Yasuda et al. 2002; Shikama and Matsuoka 2003; Bellelli et al. 2006).

### Tests of Mutational Pleiotropy

None of the mutations—singly or in combination—produced any detectable perturbations of overall secondary or tertiary structure, as measured by means of circular dichroism and UV–visible spectroscopy, respectively (supplementary fig. S4*A* and *B*, Supplementary Material online). Thus, mutational effects on the propensity for tetramer–dimer dissociation are apparently localized to intra- and interdimeric contact surfaces. The Gα4S mutation produced consistent reductions in autoxidation rate on all eight backgrounds (supplementary fig. S4*C*, Supplementary Material online) and was associated with reductions in Hb-O_2_ affinity. None of the other mutations produced a detectable change in heme autoxidation rate on any genetic background.

## Conclusion

Results of the protein engineering experiments revealed that 1/2 of all possible forward pathways connecting functionally distinct genotypes included mutational intermediates with severely compromised functional capacities. Mutational pathways that pass through these functionally aberrant intermediates appear to be selectively inaccessible, and (to the extent that *in vitro* experiments approximate *in vivo* conditions) likely represent unrealized historical pathways of functional evolution. In addition to elucidating accessibility relationships between functionally distinct ancestral and descendent genotypes, results of this analysis also demonstrate how epistasis for particular functional properties of proteins may be mediated indirectly by mutational effects on structural stability. In the case of the nightjar Hbs, sign epistasis for Hb-O_2_ affinity is indirectly attributable to mutational effects on tetramer–dimer dissociation.

## Materials and Methods

### Sample Collection and Taxonomy

We collected nightjar specimens from numerous localities in the Peruvian Andes and adjacent lowlands. All specimens were preserved as vouchers in the ornithological collection of the Museum of Southwestern Biology of the University of New Mexico and the Centro de Ornitología y Biodiversidad (CORBIDI), Lima, Peru. Complete specimen data are available via the ARCTOS online database (supplementary table S2, Supplementary Material online). All birds were live-trapped in mistnets and were sacrificed in accordance with protocols approved by the University of New Mexico Institutional Care and Use Committee (Protocol number 08UNM033-TR-100117; Animal Welfare Assurance number A4023-01). All collections were authorized by permits issued by management authorities of Peru (004-2007-INRENA-IFFS-DCB, 135-2009-AG-DGFFS-DGEFFS, 0377-2010-AG-DGFFS-DGEFFS, 0199-2012-AG-DGFFS-DGEFFS, and 006-2013-MINAGRI-DGFFS/DGEFFS).

For each nightjar specimen, we collected 20–60 μl of whole blood from the brachial or ulnar vein using heparinized microcapillary tubes. Red blood cells were separated from the plasma fraction by centrifugation, and the packed red cells were flash-frozen in liquid nitrogen. We collected pectoral muscle from each specimen as a source of both genomic DNA and globin mRNA. Muscle samples were flash-frozen or preserved using RNAlater. All tissue and blood samples were subsequently stored at −80°C. To increase taxon sampling in our survey of globin sequence variation, we obtained tissue samples for additional nightjar species from the University of Kansas Natural History Museum (*Hydropsalis maculicauda*, *H. parvula*, and *H.anomala*) and the Museum of Southwestern Biology (*Hydropsalis segmentata, Chordeiles minor, Caprimulgus rufigena, Antrostomus carolinensis, Antrostomus arizonae, Nyctipolus nigrescens, Phalaenoptilus nuttalli*, and *Nyctiphyrnus ocellatus*). Complete specimen data are provided in supplementary table S2, Supplementary Material online.

Genus and species names generally follow the South American Classification Committee (Remsen et al. 2016) and North American Classification Committee (Chesser et al. 2016) of the American Ornithologists' Union, with the exception that we adopt the broadly defined genus *Hydropsalis* as proposed by Sigurdsson and Cracraft (Sigurdsson and Cracraft 2014). Phylogeny estimates for the New World nightjars demonstrate that alternative classifications would include genera that are non-monophyletic [as would be the case for the generic names *Systellura*, *Hydropsalis*, and *Setopagis* proposed by Remsen et al. (2016)].

### Characterization of Hb Isoform Composition

To characterize Hb isoform composition in the red blood cells of the two focal nightjar species, we first separated native Hbs by means of IEF. After performing trypsin digests on excised gel bands, we then performed an MS/MS analysis to identify the resultant peptides. Database searches of MS/MS spectra were performed using Mascot (Matrix Science, v1.9.0, London, UK). Specifically, peptide mass fingerprints derived from the MS/MS analysis were used to query a custom database of avian α- and β-type globin sequences. These amino acid sequences were derived from conceptual translations of the adult-expressed α^*A*^-, α^*D*^-, and β^*A*^-globin genes of *Hydropsalis decussata* and *H. longirostris*, in addition to the full complement of embryonic and adult α- and β-type globin genes that have been annotated in the genome assemblies of other birds (Hoffmann et al. 2010, 2011; Grispo et al. 2012; Opazo et al. 2015). We identified all significant protein hits that matched more than one peptide with *P *<* *0.05. After deciphering the subunit composition of each Hb isoform by means of MS/MS, we used native gel IEF to densitometrically quantify the relative abundance of HbA and HbD (Opazo et al. 2015).

### PCR, Cloning, and Sequencing

For each of the 11 nightjar specimens used in the experimental analyses of Hb function, we extracted RNA from pectoral muscle tissue using the RNeasy kit (Qiagen,Valencia, CA) and we amplified full-length cDNAs of the α^*A*^-, α^*D*^-, and β^*A*^-globin genes using a OneStep RT-PCR kit (Qiagen, Valencia, CA). We designed paralog-specific primers using 5′ and 3′ UTR sequences from passerine species, as described previously (Opazo et al. 2015). We cloned reverse transcription (RT)-PCR products using the TOPO® TA Cloning^®^ Kit (Life technologies, Carlsbad, CA), and we sequenced at least five clones per gene in order to recover both alleles. This enabled us to determine full diploid genotypes for each of the three adult-expressed globin genes in each specimen.

We increased taxon sampling for the phylogenetic analyses by sequencing the complete coding regions of the α^*A*^-, α^*D*^-, and β^*A*^-globin genes in 13 nightjar species including *H. decussata* and *H. longirostris*. In order to maximize the number of informative characters in our phylogenetic analysis of the α^*A*^-globin gene, we used genomic DNA templates for all 13 species to sequence the complete coding region in addition to the introns and noncoding flanking regions. We extracted DNA from pectoral muscle tissue using the DNeasy kit (Qiagen, Valencia, CA).

### Phylogenetic Analysis

We estimated phylogenies of the α- and β-type globin genes using maximum likelihood, with support for internal nodes assessed with 500 bootstrap replicates. We estimated the trees using PhyML (Guindon et al. 2010), with the HKY85 substitution model and added parameters for rate heterogeneity among sites (modeled according to a gamma distribution with four rate categories) and invariant sites.

### Protein Purification and *In Vitro* Analysis of Hb Function

We purified HbA and HbD from hemolysates of wildcaught *H. decussata* and *H. longirostris* specimens by means of anion-exchange fast-protein liquid chromatography (FPLC) using an Äkta Pure system and a HiTrap QHP column (GE Healthcare). This procedure also removes endogenous organic phosphates, yielding “stripped” Hb samples. Using purified Hb solutions (0.3 mM heme), we measured O_2_-equilibrium curves at 37 °C in 0.1 M HEPES buffer (pH 7.4) in the absence (stripped) and presence of 0.1 M KCl and IHP (at 2-fold molar excess over tetrameric Hb), and in the simultaneous presence of KCl and IHP. We measured O_2_-equilibria of 3 μl thin-film samples in a modified diffusion chamber where absorption at 436 nm was monitored during stepwise changes in the equilibration of N_2_/O_2_ mixtures generated by precision Wösthoff gas-mixing pumps (Weber 1992). We estimated values of *P*_50_ and *n*_50_ (Hill’s cooperativity coefficient at half-saturation) by fitting the Hill equation *Y *=* P*$O_{2}^{n}$/(*P*_50_^*n *^+*^ ^P*$O_{2}^{n}$) to the experimental O_2_ saturation data by means of nonlinear regression (*Y *=* *fractional O_2_ saturation; *n*, cooperativity coefficient) (Grispo et al. 2012; Weber et al. 2013). The nonlinear fitting was based on 5–8 equilibration steps between 30% and 70% oxygenation. Free Cl^−^ concentrations were measured with a model 926S Mark II chloride analyzer (Sherwood Scientific Ltd, Cambridge, UK).

### Vector Construction and Site-Directed Mutagenesis

The α^*A*^- and β^*A*^-globin sequences were synthesized by Eurofins MWG Operon (Huntsville, AL, USA) after optimizing the nucleotide sequences in accordance with *E. coli* codon preferences. The synthesized α^*A*^–β^*A*^ globin gene cassette was cloned into a custom pGM vector system along with the *methionine aminopeptidase* (MAP) gene, as described previously (Natarajan et al. 2011, 2013). We engineered each of the α- and β-chain codon substitutions using the QuikChange® II XL Site-Directed Mutagenesis kit from Stratagene (La Jolla, CA, USA). Each engineered codon change was verified by DNA sequencing.

### Expression and Purification of Recombinant Hbs

We carried out recombinant Hb expression in the JM109 (DE3) *E. coli* strain. Bacterial cells were selected in LB agar with dual antibiotics (ampicillin and kanamycin) to ensure that transformants received both pGM and pCO-MAP plasmids for expression. The expression of each rHb mutant was carried out in 1.5 L of TB medium. Bacterial cells were grown in 37°C in an orbital shaker at 200 rpm until absorbance values reached 0.6–0.8 at 600 nm. The bacterial cultures were induced by 0.2 mM IPTG and were then supplemented with hemin (50 μg/ml) and glucose (20 g/l). The bacterial culture conditions and the protocol for preparing cell lysates are described in Natarajan et al. (2011).

We purified each rHb sample by means of two step ion-exchange chromatography as described previously (Natarajan et al. 2011, 2013; Projecto-Garcia et al. 2013; Cheviron et al. 2014; Galen et al. 2015; Natarajan et al. 2015b; Tufts et al. 2015; Natarajan et al. 2016). Samples were passed through an anion-exchange column (HiTrap™ Q-XL, GE Healthcare, 17-5159-01), equilibrated with 20 mM CAPS buffer (0.5 mM EDTA, pH 9.7), and eluted using a linear gradient of 0–1.0 M NaCl. The eluted fractions were passed through a cation-exchange column (HiTrap^TM^ SP-XL, GE Healthcare, 17-5161-01) equilibrated with 20 mM sodium phosphate buffer (0.5 mM EDTA, pH 7.2); the samples were then eluted using a linear gradient of 10 mM sodium phosphate buffer (0.5 mM EDTA, pH 9.0). The samples were desalted by dialysis against 10 mM HEPES buffer (pH 7.4) at 4 °C. The eluted fractions of each rHb sample were concentrated by means of centrifugal filtration. The purified rHb samples were analyzed by sodium dodecyl sulphate (SDS)-polyacrylamide gel electrophoresis (supplementary fig. S5, Supplementary Material online) and IEF, which confirmed the absence of subunit heterogeneity in the analyzed rHb samples. After preparing rHb samples as oxyHb, deoxyHb, and carbonmonoxy derivatives, we measured absorbance at 450–600 nm to confirm that the absorbance maxima match those of the native HbA samples.

### Measures of Tetramer–Dimer Equilibria

We tested for evidence of tetramer–dimer dissociation by means of gel filtration chromatography using FPLC (ÄKTA Pure, GE Healthcare). We used a Superdex 75 10/300 GL column that was pre-equilibrated with 50mM potassium phosphate buffer, pH 7.0, 0.5 mM EDTA, 0.15 M NaCl, and we used horse myoglobin and human Hb as protein markers. Human Hb was used as a marker for tetrameric assembly, but it should be noted that vertebrate Hbs typically show apparent molecular weights <64 kDa due to reversible dissociation into dimers during passage through the chromatography medium (Weber et al. 2013; Storz et al. 2015). Using the linear relationship between elution volume and log-transformed molecular weight for the reference proteins, we calculated the log-molecular weight of each nightjar rHb mutant by solving the regression equation *y* = 0.330*x* + 5.329, where *x* is the measured elution volume. We prepared the protein samples in the same buffer using a heme concentration of 0.16 mM. We measured absorbance at 415 nm with a flow rate of 0.5 ml/min.

### Spectroscopic Measurements of Structural Stability

We assessed the pH-dependent stability of the 16 rHbs by means of UV–visible spectroscopy. We prepared 20 mM filtered buffers spanning the pH range 2.0–11.0. We prepared 20 mM glycine-HCl for pH 2.0–3.5; 20 mM acetate for pH 4.0–5.5; 20 mM phosphate for pH 6.0–8.0; 20 mM glycine-NaOH for pH 8.5–10.0; 20 mM carbonate-NaOH for pH 10.5 and phosphate-NaOH for pH 11.0. We diluted the purified rHb samples in the pH-specific buffers to achieve uniform protein concentrations of 0.15 mg/ml. We incubated the samples for 3–4 h at 25 °C prior to spectroscopic measurements, and we maintained this same temperature during the course of the experiments. We measured absorbance in the range 260–700 nm using a Cary Varian Bio100 UV–Vis spectrophotometer (Varian Inc., USA) with Quartz cuvettes, and we used IGOR Pro 6 (WaveMetrics Inc., USA) to process the raw spectra. For the same set of 16 rHb mutants, we tested for changes in secondary structure of the globin chains by measuring circular dichroism spectra on a JASCO J-815 spectropolarimeter (JASCO Corp., Japan) using a quartz cell with a path length of 1 mm. We assessed changes in secondary structure by measuring molar ellipticity in the far UV region between 190 and 260 nm in three consecutive spectral scans per sample.

### Measurement of Autoxidation Rate

We treated purified rHb samples with potassium ferricyanide (K_3_[Fe(CN)_6_]) to remove bound CO, and we maintained rHbs in the ferrous (Fe^2+^) state by treating the samples with sodium dithionite (Na_2_S_2_O_4_). We then prepared oxyHb by passing the samples through a Sephadex G-50 column. For each rate measurement, we used 200 μl of 20 µM oxyHb in 100 mM potassium phosphate buffer, pH 7.0, containing 1 mM EDTA and 3 mmol/mol of heme catalase and superoxide dismutase. To measure the spontaneous conversion of ferrous (Fe^2+^) oxyHb to ferric (Fe^3+^) metHb we recorded the absorbance spectrum at regular intervals over a 90 h period. The spectra were collected between 400 and 700 nm using a BioTek Synergy2 multi-mode microplate reader (BioTek Instruments, Inc., USA). We estimated autoxidation rates by plotting the A_541_/A_630_ ratio (ratio of absorbances at 540 and 630 nm) vs. time, using IGOR Pro 6.37 software (Wavemetrics, Inc., USA).

### Structural Modeling

We modeled structures of nightjar Hbs using MODELLER ver. 9.17 (Webb and Sali 2016). We selected human Hb (DPB ID 1hho and 3hhb) as templates for oxy- and deoxyHb conformations. We performed additional calculations using Hydrogen Bond Calculation Web server ver. 1.1 (http://cib.cf.ocha.ac.jp/bitool/HBOND/) and the PyMOL Molecular Graphics System (ver. 1.8; Schrödinger, San Diego, CA).

## Supplementary Material

Supplementary data are available at *Molecular Biology and Evolution* online.

## Supplementary Material

Supplementary Data

## References

[msx085-B1] Adams JG, Boxer LA, Baehner RL, Forget BG, Tsistrakis GA, Steinberg MH. 1979 Hemoglobin Indianapolis (β112[G14] arginine). An unstable β-chain variant producing the phenotype of severe β-thalassemia. J Clin Invest. 63:931–938. 447835 10.1172/JCI109393 PMC372034

[msx085-B2] Arnone A, Perutz MF. 1974 Structure of inositol-hexaphosphate-human deoxyhaemoglobin complex. Nature 249:34–36. 4364353 10.1038/249034a0

[msx085-B3] Baldwin J, Chothia C. 1979 Haemoglobin: the structural changes related to ligand binding and its allosteric mechanism. J Mol Biol. 129:175–220. 39173 10.1016/0022-2836(79)90277-8

[msx085-B4] Bank C, Matuszewski S, Hietpas RT, Jensen JD. 2016 On the (un)predictability of a large intragenic fitness landscape. Proc Natl Acad Sci U S A. 113:14085–14090. 27864516 10.1073/pnas.1612676113 PMC5150413

[msx085-B5] Bellelli A, Brunori M, Miele AE, Panetta G, Vallone B. 2006 The allosteric properties of hemoglobin: insights from natural and site directed mutants. Curr Protein Pept Sci. 7:17–45. 16472167 10.2174/138920306775474121

[msx085-B6] Benham PM, Beckman EJ, DuBay SG, Monica Flores L, Johnson AB, Lelevier MJ, Schmitt CJ, Wright NA, Witt CC. 2011 Satellite imagery reveals new critical habitat for endangered bird species in the high Andes of Peru. Endanger Species Res. 13:145–157.

[msx085-B7] Bloom JD, Arnold FH. 2009 In the light of directed evolution: pathways of adaptive protein evolution. Proc Natl Acad Sci U S A. 106:9995–10000. 19528653 10.1073/pnas.0901522106 PMC2702793

[msx085-B8] Brennan SO, Potter HC, Kubala LM, Carnoutsos SA, Ferguson MM. 2002 Hb Canterbury [β112(G14)Cys→Phe]: a new, mildly unstable variant. Hemoglobin 26:67–69. 11939514 10.1081/hem-120002942

[msx085-B9] Bridgham JT, Carroll SM, Thornton JW. 2006 Evolution of hormone-receptor complexity by molecular exploitation. Science 312:97–101. 37000978 10.1681/01.asn.0000926836.46869.e5

[msx085-B10] Bridgham JT, Ortlund EA, Thornton JW. 2009 An epistatic ratchet constrains the direction of glucocorticoid receptor evolution. Nature 461:515–519. 19779450 10.1038/nature08249 PMC6141187

[msx085-B11] Brown KM, Costanzo MS, Xu W, Roy S, Lozovsky ER, Hartl DL. 2010 Compensatory mutations restore fitness during the evolution of dihydrofolate reductase. Mol Biol Evol. 27:2682–2690. 20576759 10.1093/molbev/msq160 PMC2981517

[msx085-B12] Carneiro M, Hartl DL. 2010 Adaptive landscapes and protein evolution. Proc Natl Acad Sci U S A. 107:1747–1751. 19805125 10.1073/pnas.0906192106 PMC2868285

[msx085-B13] Carroll SM, Ortlund EA, Thornton JW. 2011 Mechanisms for the evolution of a derived function in the ancestral glucocorticoid receptor. PLoS Genet. 7:e1002117. 21698144 10.1371/journal.pgen.1002117 PMC3116920

[msx085-B14] Chang CK, Simplaceanu V, Ho C. 2002 Effects of amino acid substitutions at β131 on the structure and properties of hemoglobin: evidence for communication between α_1_β_1_- and α_1_β_2_-subunit interfaces. Biochemistry 41:5644–5655. 11969426 10.1021/bi011919d

[msx085-B15] Chesser RT, Burns KJ, Cicero C, Dunn JL, Kratter AW, Lovette IJ, Rasmussen PC, Remsen JV, Rising JD, Stotz DF, 2016 Fifty-seventh supplement to the American Ornithologists' Union Check-list of North American Birds. Auk 133:544–560.

[msx085-B16] Cheviron ZA, Natarajan C, Projecto-Garcia J, Eddy DK, Jones J, Carling MD, Witt CC, Moriyama H, Weber RE, Fago A, 2014 Integrating evolutionary and functional tests of adaptive hypotheses: a case study of altitudinal differentiation in hemoglobin function in an Andean sparrow, *Zonotrichia capensis*. Mol Biol Evol. 31:2948–2962. 25135942 10.1093/molbev/msu234 PMC4209134

[msx085-B17] Covert AW, Lenski RE, Wilke CO, Ofria C. 2013 Experiments on the role of deleterious mutations as stepping stones in adaptive evolution. Proc Natl Acad Sci U S A. 110:E3171–E3178. 23918358 10.1073/pnas.1313424110 PMC3752215

[msx085-B18] da Silva J, Coetzer M, Nedellec R, Pastore C, Mosier DE. 2010 Fitness epistasis and constraints on adaptation in a human immunodeficiency virus type 1 protein region. Genetics 185:293–303. 20157005 10.1534/genetics.109.112458 PMC2870964

[msx085-B19] de Visser JAGM, Krug J. 2014 Empirical fitness landscapes and the predictability of evolution. Nat Rev Genet. 15:480–490. 24913663 10.1038/nrg3744

[msx085-B20] DePristo MA, Hartl DL, Weinreich DM. 2007 Mutational reversions during adaptive protein evolution. Mol Biol Evol. 24:1608–1610. 17556755 10.1093/molbev/msm118

[msx085-B21] DePristo MA, Weinreich DM, Hartl DL. 2005 Missense meanderings in sequence space: a biophysical view of protein evolution. Nat Rev Genet 6:678–687. 16074985 10.1038/nrg1672

[msx085-B22] Dickinson BC, Leconte AM, Allen B, Esvelt KM, Liu DR. 2013 Experimental interrogation of the path dependence and stochasticity of protein evolution using phage-assisted continuous evolution. Proc Natl Acad Sci U S A. 110:9007–9012. 23674678 10.1073/pnas.1220670110 PMC3670371

[msx085-B23] Franke J, Kloezer A, de Visser JAGM, Krug J. 2011 Evolutionary accessibility of mutational pathways. PLoS Comput Biol. 7:e1002134. 21876664 10.1371/journal.pcbi.1002134 PMC3158036

[msx085-B24] Fronticelli C, Gattoni M, Lu AL, Brinigar WS, Bucci JLG, Chiancone E. 1994 The dimer-tetramer equilibrium of recombinant hemoglobins—stabilization of the α_1_β_2_ interface by the mutation β(Cys112→Gly) at the α_1_β_1_ interface. Biophys Chem. 51:53–57. 8061226 10.1016/0301-4622(94)00028-x

[msx085-B25] Galen SC, Natarajan C, Moriyama H, Weber RE, Fago A, Benham PM, Chavez AN, Cheviron ZA, Storz JF, Witt CC. 2015 Contribution of a mutational hotspot to adaptive changes in hemoglobin function in high-altitude Andean house wrens. Proc Natl Acad Sci U S A. 112:13958–13963. 26460028 10.1073/pnas.1507300112 PMC4653164

[msx085-B26] Gong LI, Suchard MA, Bloom JD. 2013 Stability-mediated epistasis constrains the evolution of an influenza protein. eLife 2:e00631. 23682315 10.7554/eLife.00631 PMC3654441

[msx085-B27] Grispo MT, Natarajan C, Projecto-Garcia J, Moriyama H, Weber RE, Storz JF. 2012 Gene duplication and the evolution of hemoglobin isoform differentiation in birds. J Biol Chem. 287:37647–37658. 22962007 10.1074/jbc.M112.375600 PMC3488042

[msx085-B28] Guindon S, Dufayard JF, Lefort V, Anisimova M, Hordijk W, Gascuel O. 2010 New algorithms and methods to estimate maximum-likelihood phylogenies: assessing the performance of PhyML 3.0. Syst Biol. 59:307–321. 20525638 10.1093/sysbio/syq010

[msx085-B29] Han KL, Robbins MB, Braun MJ. 2010 A multi-gene estimate of phylogeny in the nightjars and nighthawks (Caprimulgidae). Mol Phylogenet Evol. 55:443–453. 20123032 10.1016/j.ympev.2010.01.023

[msx085-B30] Harms MJ, Thornton JW. 2013 Evolutionary biochemistry: revealing the historical and physical causes of protein properties. Nat Rev Genet. 14:559–571. 23864121 10.1038/nrg3540 PMC4418793

[msx085-B31] Harms MJ, Thornton JW. 2014 Historical contingency and its biophysical basis in glucocorticoid receptor evolution. Nature 512:203–207. 24930765 10.1038/nature13410 PMC4447330

[msx085-B32] Hartl DL. 2014 What can we learn from fitness landscapes? Curr Opin Microbiol. 21:51–57. 25444121 10.1016/j.mib.2014.08.001 PMC4254422

[msx085-B33] Hoffmann FG, Opazo JC, Storz JF. 2011 Differential loss and retention of cytoglobin, myoglobin, and globin-E during the radiation of vertebrates. Genome Biol Evol. 3:588–600. 21697098 10.1093/gbe/evr055 PMC3156568

[msx085-B34] Hoffmann FG, Storz JF. 2007 The α*D*-globin gene originated via duplication of an embryonic α-like globin gene in the ancestor of tetrapod vertebrates. Mol Biol Evol. 24:1982–1990. 17586601 10.1093/molbev/msm127

[msx085-B35] Hoffmann FG, Storz JF, Gorr TA, Opazo JC. 2010 Lineage-specific patterns of functional diversification in the α- and β-globin gene families of tetrapod vertebrates. Mol Biol Evol. 27:1126–1138. 20047955 10.1093/molbev/msp325 PMC2877528

[msx085-B36] Imai K. 1982 Allosteric effects in haemoglobin. Cambridge: Cambridge University Press.

[msx085-B37] Janecka JE, Nielsen SSE, Andersen SD, Hoffmann FG, Weber RE, Anderson T, Storz JF, Fago A. 2015 Genetically based low oxygen affinities of felid hemoglobins: lack of biochemical adaptation to high-altitude hypoxia in the snow leopard. J Exp Biol. 218:2402–2409. 26246610 10.1242/jeb.125369 PMC4528707

[msx085-B38] Kimura M. 1985 The role of compensatory neutral mutations in molecular evolution. J Genet. 64:7–19.

[msx085-B39] Kondrashov DA, Kondrashov FA. 2015 Topological features of rugged fitness landscapes in sequence space. Trends Genet. 31:24–33. 25438718 10.1016/j.tig.2014.09.009

[msx085-B40] Kvitek DJ, Sherlock G. 2011 Reciprocal sign epistasis between frequently experimentally evolved adaptive mutations causes a rugged fitness landscape. PLoS Genet. 7:e1002056. 21552329 10.1371/journal.pgen.1002056 PMC3084205

[msx085-B41] Larsen C, Speed M, Harvey N, Noyes HA. 2007 A molecular phylogeny of the nightjars (Aves: Caprimulgidae) suggests extensive conservation of primitive morphological traits across multiple lineages. Mol Phylogenet Evol. 42:789–796. 17123840 10.1016/j.ympev.2006.10.005

[msx085-B42] Lozovsky ER, Chookajorn T, Brown KM, Imwong M, Shaw PJ, Kamchonwongpaisan S, Neafsey DE, Weinreich DM, Hartl DL. 2009 Stepwise acquisition of pyrimethamine resistance in the malaria parasite. Proc Natl Acad Sci U S A. 106:12025–12030. 19587242 10.1073/pnas.0905922106 PMC2715478

[msx085-B43] Lunzer M, Milter SP, Felsheim R, Dean AM. 2005 The biochemical architecture of an ancient adaptive landscape. Science 310:499–501. 16239478 10.1126/science.1115649

[msx085-B44] Mairbäurl H, Weber RE. 2012 Oxygen transport by hemoglobin. Compr Physiol. 2:1463–1489. 23798307 10.1002/cphy.c080113

[msx085-B45] Malcolm BA, Wilson KP, Matthews BW, Kirsch JF, Wilson AC. 1990 Ancestral lysozymes reconstructed, neutrality tested, and thermostability linked to hydrocarbon packing. Nature 345:86–89. 2330057 10.1038/345086a0

[msx085-B46] Maynard Smith J. 1970 Natural selection and the concept of a protein space. Nature 225:563–564. 5411867 10.1038/225563a0

[msx085-B47] McCandlish DM, Shah P, Plotkin JB. 2016 Epistasis and the dynamics of reversion in molecular evolution. Genetics 203:1335–1351. 27194749 10.1534/genetics.116.188961 PMC4937490

[msx085-B48] Meer MV, Kondrashov AS, Artzy-Randrup Y, Kondrashov FA. 2010 Compensatory evolution in mitochondrial tRNAs navigates valleys of low fitness. Nature 464:279–282. 20182427 10.1038/nature08691

[msx085-B49] Miton CM, Tokuriki N. 2016 How mutational epistasis impairs predictability in protein evolution and design. Protein Sci. 25:1260–1272. 26757214 10.1002/pro.2876 PMC4918425

[msx085-B50] Natarajan C, Hoffman FG, Lanier HC, Wolf CJ, Cheviron ZA, Spangler ML, Weber RE, Fago A, Storz JF. 2015a Intraspecific polymorphism, interspecific divergence, and the origins of function-altering mutations in deer mouse hemoglobin. Mol Biol Evol. 32:978–997. 25556236 10.1093/molbev/msu403 PMC4379404

[msx085-B51] Natarajan C, Hoffmann FG, Weber RE, Fago A, Witt CC, Storz JF. 2016 Predictable convergence in hemoglobin function has unpredictable molecular underpinnings. Science 354:336–340. 27846568 10.1126/science.aaf9070 PMC5464326

[msx085-B52] Natarajan C, Inoguchi N, Weber RE, Fago A, Moriyama H, Storz JF. 2013 Epistasis among adaptive mutations in deer mouse hemoglobin. Science 340:1324–1327. 23766324 10.1126/science.1236862 PMC4409680

[msx085-B53] Natarajan C, Jiang X, Fago A, Weber RE, Moriyama H, Storz JF. 2011 Expression and purification of recombinant hemoglobin in *Escherichia coli*. PLoS One 6:e20176. 21625463 10.1371/journal.pone.0020176 PMC3098879

[msx085-B54] Natarajan C, Projecto-Garcia J, Moriyama H, Weber RE, Munoz-Fuentes V, Green AJ, Kopuchian C, Tubaro PL, Alza L, Bulgarella M, 2015b Convergent evolution of hemoglobin function in high-altitude Andean waterfowl involves limited parallelism at the molecular sequence level. PLoS Genet. 11:e1005681. 10.1371/journal.pgen.1005681 PMC4670201 26637114

[msx085-B55] Opazo JC, Hoffman FG, Natarajan C, Witt CC, Berenbrink M, Storz JF. 2015 Gene turnover in the avian globin gene family and evolutionary changes in hemoglobin isoform expression. Mol Biol Evol. 32:871–887. 25502940 10.1093/molbev/msu341 PMC4379397

[msx085-B56] Palmer AC, Toprak E, Baym M, Kim S, Veres A, Bershtein S, Kishony R. 2015 Delayed commitment to evolutionary fate in antibiotic resistance fitness landscapes. Nat Commun. 6:7385. 26060115 10.1038/ncomms8385 PMC4548896

[msx085-B57] Perutz MF. 1989 Mechanisms of cooperativity and allosteric regulation in proteins. Q Rev Biophys. 22:139–236. 2675171 10.1017/s0033583500003826

[msx085-B58] Pettigrew DW, Romeo PH, Tsapis A, Thillet J, Smith ML, Turner BW, Ackers GK. 1982 Probing the energetics of proteins through structural perturbation—sites of regulatory energy in human hemoglobin. Proc Natl Acad Sci U S A. 79:1849–1853. 6952235 10.1073/pnas.79.6.1849 PMC346078

[msx085-B59] Poelwijk FJ, Kiviet DJ, Weinreich DM, Tans SJ. 2007 Empirical fitness landscapes reveal accessible evolutionary paths. Nature 445:383–386. 17251971 10.1038/nature05451

[msx085-B60] Projecto-Garcia J, Natarajan C, Moriyama H, Weber RE, Fago A, Cheviron ZA, Dudley R, McGuire JA, Witt CC, Storz JF. 2013 Repeated elevational transitions in hemoglobin function during the evolution of Andean hummingbirds. Proc Natl Acad Sci U S A. 110:20669–20674. 24297909 10.1073/pnas.1315456110 PMC3870697

[msx085-B61] Remsen JV, Areta JI, Cadena CD, Claramunt S, Jaramillo A, Pacheco JF, Perez-Eman J, Robbins MB, Stiles FG, Stotz DF, 2016 A classification of the bird species of South America. American Ornithologists' Union. http://www.museum.lsu.edu/∼Remsen/SACCBaseline.htm.

[msx085-B62] Revsbech IG, Tufts DM, Projecto-Garcia J, Moriyama H, Weber RE, Storz JF, Fago A. 2013 Hemoglobin function and allosteric regulation in semi-fossorial rodents (family Sciuridae) with different altitudinal ranges. J Exp Biol. 216:4264–4271. 24172889 10.1242/jeb.091397 PMC3813580

[msx085-B63] Salverda MLM, Dellus E, Gorter FA, Debets AJM, van der Oost J, Hoekstra RF, Tawfik DS, de Visser JAGM. 2011 Initial mutations direct alternative pathways of protein evolution. PLoS Genet. 7:e1001321. 21408208 10.1371/journal.pgen.1001321 PMC3048372

[msx085-B64] Schenk MF, Szendro IG, Salverda MLM, Krug J, de Visser JAGM. 2013 Patterns of epistasis between beneficial mutations in an antibiotic resistance gene. Mol Biol Evol. 30:1779–1787. 23676768 10.1093/molbev/mst096 PMC3708503

[msx085-B65] Schulenberg TS, Stotz DF, Lane DF, O'Neill JP, Parker TA. 2007 Birds of Peru. Princeton, NJ: Princeton University Press.

[msx085-B66] Shikama K, Matsuoka A. 2003 Human haemoglobin—a new paradigm for oxygen binding involving two types of αβ contacts. Eur J Biochem. 270:4041–4051. 14519115 10.1046/j.1432-1033.2003.03791.x

[msx085-B67] Sigurdsson S, Cracraft J. 2014 Deciphering the diversity and history of New World nightjars (Aves: Caprimulgidae) using molecular phylogenetics. Zool J Linn Soc. 170:506–545.

[msx085-B68] Starr TN, Thornton JW. 2016 Epistasis in protein evolution. Protein Sci. 25:1204–1218. 26833806 10.1002/pro.2897 PMC4918427

[msx085-B69] Steinberg MH, Nagel RL. 2009 Unstable hemoglobins, hemoglobins with altered oxygen affinity, Hemoglobin M, and other variants of clinical and biological interest In: Steinberg MH, Forget BG, Higgs DR, Weatherall DJ, editors. Disorders of hemoglobin: genetics, pathophysiology, and clinical management. 2nd edn Cambridge: Cambridge University Press p. 589–606.

[msx085-B70] Storz JF. 2016a Causes of molecular convergence and parallelism in protein evolution. Nat Rev Genet. 17:239–250. 26972590 10.1038/nrg.2016.11 PMC5482790

[msx085-B71] Storz JF. 2016b Hemoglobin-oxygen affinity in high-altitude vertebrates: is there evidence for an adaptive trend? J Exp Biol. 219:3190–3203. 27802149 10.1242/jeb.127134 PMC5091379

[msx085-B72] Storz JF, Natarajan C, Moriyama H, Hoffmann FG, Wang T, Fago A, Malte H, Overgaard J, Weber RE. 2015 Oxygenation properties and isoform diversity of snake hemoglobins. Am J Physiol Regul Integr Comp Physiol. 309:R1178–R1191. 26354849 10.1152/ajpregu.00327.2015 PMC4666957

[msx085-B73] Storz JF, Runck AM, Moriyama H, Weber RE, Fago A. 2010 Genetic differences in hemoglobin function between highland and lowland deer mice. J Exp Biol. 213:2565–2574. 20639417 10.1242/jeb.042598 PMC2905302

[msx085-B74] Storz JF, Runck AM, Sabatino SJ, Kelly JK, Ferrand N, Moriyama H, Weber RE, Fago A. 2009 Evolutionary and functional insights into the mechanism underlying high-altitude adaptation of deer mouse hemoglobin. Proc Natl Acad Sci U S A. 106:14450–14455. 19667207 10.1073/pnas.0905224106 PMC2732835

[msx085-B75] Tsuruga M, Matsuoka A, Hachimori A, Sugawara Y, Shikama K. 1998 The molecular mechanism of autoxidation for human oxyhemoglobin—tilting of the distal histidine causes nonequivalent oxidation in the beta chain. J Biol Chem. 273:8607–8615. 9535834 10.1074/jbc.273.15.8607

[msx085-B76] Tufts DM, Natarajan C, Revsbech IG, Projecto-Garcia J, Hoffman FG, Weber RE, Fago A, Moriyama H, Storz JF. 2015 Epistasis constrains mutational pathways of hemoglobin adaptation in high-altitude pikas. Mol Biol Evol. 32:287–298. 25415962 10.1093/molbev/msu311 PMC4298171

[msx085-B77] Vasquez GB, Karavitis M, Ji XH, Pechik I, Brinigar WS, Gilliland GL, Fronticelli C. 1999 Cysteines β93 and β112 as probes of conformational and functional events at the human hemoglobin subunit interfaces. Biophys J. 76:88–97. 9876125 10.1016/S0006-3495(99)77180-8 PMC1302502

[msx085-B78] Wagner A. 2008 Neutralism and selectionism: a network-based reconciliation. Nat Rev Genet. 9:965–974. 18957969 10.1038/nrg2473

[msx085-B79] Wajcman H, Galactéros F. 1996 Abnormal hemoglobins with high oxygen affinity and erythrocytosis. Hematol Cell Ther. 38:305–312. 8891722 10.1007/s00282-996-0305-4

[msx085-B80] Webb B, Sali A. 2016 Comparative protein structure modeling using MODELLER. Curr Protoc Bioinformatics 54:5.6.1–5.6.37. 10.1002/cpbi.3 PMC5031415 27322406

[msx085-B81] Weber RE. 1992 Use of ionic and zwitterionic (tris bistris and HEPES) buffers in studies on hemoglobin function. J Appl Physiol. 72:1611–1615. 1592755 10.1152/jappl.1992.72.4.1611

[msx085-B82] Weber RE, Fago A, Malte H, Storz JF, Gorr TA. 2013 Lack of conventional oxygen-linked proton and anion binding sites does not impair allosteric regulation of oxygen binding in dwarf caiman hemoglobin. Am J Physiol Regul Integr Comp Physiol. 305:R300–R312. 23720132 10.1152/ajpregu.00014.2013 PMC3743003

[msx085-B83] Weinreich DM. 2010 Predicting molecular evolutionary trajectories in principle and practice. In: Encyclopedia of life sciences. Chichester: John Wiley & Sons Ltd. doi: 10.1002/9780470015902.a9780470022174.

[msx085-B84] Weinreich DM, Chao L. 2005 Rapid evolutionary escape by large populations from local fitness peaks is likely in nature. Evolution 59:1175–1182. 16050095

[msx085-B85] Weinreich DM, Delaney NF, DePristo MA, Hartl DL. 2006 Darwinian evolution can follow only very few mutational paths to fitter proteins. Science 312:111–114. 16601193 10.1126/science.1123539

[msx085-B86] Weinreich DM, Lan Y, Wylie CS, Heckendorn RB. 2013 Should evolutionary geneticists worry about higher-order epistasis? Curr Opin Genet Dev. 23:700–707. 24290990 10.1016/j.gde.2013.10.007 PMC4313208

[msx085-B87] Weinreich DM, Watson RA, Chao L. 2005 Sign epistasis and genetic constraint on evolutionary trajectories. Evolution 59:1165–1174. 16050094

[msx085-B88] Wu NC, Dai L, Olson CA, Lloyd-Smith JO, Sun R. 2016 Adaptation in protein fitness landscapes is facilitated by indirect paths. eLife 5:e16965. 27391790 10.7554/eLife.16965 PMC4985287

[msx085-B89] Yasuda JP, Ichikawa T, Tsuruga M, Matsuoka A, Sugawara Y, Shikama K. 2002 The α_1_β_1_ contact of human hemoglobin plays a key role in stabilizing the bound dioxygen—further evidence from the iron valency hybrids. Eur J Biochem. 269:202–211. 11784314 10.1046/j.0014-2956.2002.02635.x

