# Supplementary material for: Stability-Mediated Epistasis Restricts Accessible Mutational Pathways in the Functional Evolution of Avian Hemoglobin
Source: Mol Biol Evol. 2017 Feb 13;34(5):1240–51. doi: 10.1093/molbev/msx085 (PMC5400398; doi:10.1093/molbev/msx085)
Supplement: Supplementary Data [file msx085_Supp.pdf]

Supplemental Material

Kumar *et al.*

Legends for Supplemental Figures

Fig. S1. Native IEF gel images showing Hb isoform composition of red cell lysates from South American nightjars. A) Coomassie-stained IEF gel (pH 3–9) showing the separation of native HbA and HbD isoforms from red cell lysates of *Hydropsalis longirostris* (left) and *H. decussata* (right). Specimen identification numbers are given for each sample and 'M' denotes the IEF standard with bands corresponding to isoelectric points 3-10. B) Unstained gel image with red bands representing native HBA and HBD isoforms.

Fig. S2. Phylogenies of α^{A}- and β^{A}-globin genes in nightjars (family Caprimulgidae) reveal the polarity of character state changes at each of the four amino acid sites that distinguish the HbA isoforms of the two focal species, *Hydropsalis longirostris* and *H. decussata*. Phylogenies were estimated using maximum likelihood; percent bootstrap support is indicated at each node. Two hummingbird species were used as outgroups (gray branches). Alignments of amino acid sites that distinguish Hbs of the two species are shown in Fig. 2. Genealogical discordance between the two trees likely reflects incomplete lineage sorting and/or introgressive hybridization. (*A*) Estimated phylogeny of α^{A}-globin orthologs from 13 species of New World nightjars, based on alignments of nucleotide sequence. The Aα34V substitution occurred in the common ancestor of the *H. decussata* and *H. segmentata* orthologs, and the Gα4S and Vα13I substitutions occurred subsequently in the *decussata*-specific branch. (*B*) Estimated phylogeny of the β^{A}-globin gene in 12 of same 13 nightjar species. The Iβ112V substitution clearly occurred in the external branch leading to the *H. decussata* ortholog.

Fig. S3. The two substitutions in the α-chain A-helix, Gα4S and Vα13I, are predicted to reduce Hb-O_2 affinity by reducing the relative stability of the R-state. (*A*) In oxyHb, the carbonyl oxygen of α4G (located at the N-terminus of the A-helix) forms two hydrogen bonds with α4T, one with the γ1-oxygen and one with the main-chain nitrogen; the same contact is present in deoxyHb (not shown). (*B*) The Gα4S mutation eliminates the α4-α8T contact only in oxyHb, thereby destabilizing the R-state relative to the T-state. (*C*) In oxyHb, the γ2-carbon of α13V forms a weak van der Waals contact with the δ1-carbon of α125L. (*D*) Replacing Val with Ile at α13 increases the rigidity of the A-helix because the δ1-carbon of Ile forms a stronger hydrophobic contact with α125L via interactions with both the δ1- and δ2-carbons of the Leu side chain. In deoxyHb, the same hydrophobic contacts between α13V/I and α125L do not exist (not shown). By conferring an added rigidity to the A-helix of oxyHb, the Vα13I mutation weakens the bonds between the ζ-carbon of the adjacent α14F and the γ1- and γ2-carbons of α67V. This point of contact between α14F and α67V serves as a hinge for allosteric motion, so constraints on that motion imposed by the Vα13I mutation are predicted to reduce Hb-O_2 affinity by increasing the free energy of the oxygenation-linked T→R transition in quaternary structure.

Fig. S4. Tests of biophysical and biochemical properties of the 16 rHbs (the ancestral GVAI genotype, the quadruple-mutant SIVV genotype, and all possible mutational intermediates that connect them). (*A*) UV-visible spectroscopy revealed no detectable variation in the integrity of globin chain tertiary structure or heme retention among the 16 rHbs, as illustrated by pH titration profiles in the pH range 2.0-11.0 (measured at intervals of 0.5 pH units). We measured the absorbance of each rHb at Soret peak (415 nm). (*B*) Circular Dichroism spectroscopy revealed no detectable variation in the integrity of globin chain secondary structure (α-helicity), as illustrated by pH titration profiles in the pH range 2.0-11.0 (measured

at intervals of 0.5 pH units). We measured ellipticities at 222 nm. (*C*) The Gα4S mutation was strongly associated with a reduced autoxidation rate (the rate at which ferrous [Fe^{2+}] oxyHb is oxidized to the ferric [Fe^{3+}] form in air). Autoxidation rates for Hbs with the derived α4-Ser and the ancestral α4-Gly were 0.003-0.010 h^{-1} and 0.015-0.025 h^{-1}, respectively. For each rHb, we spectroscopically measured the ratio of absorbance at 541nm (QIII band) to 630nm (QI band). We then estimated rates from plots of the A_{541}/A_{630} ratio (normalized to 1.0) vs time. Aside from Gα4S, none of the other mutations showed any significant association with autoxidation rate.

Fig. S5. 20% SDS-PAGE gel image showing the purified fractions of recombinantly expressed nightjar Hb representing the 'SIVV' genotype (the wildtype genotype of *H. decussata*). Lane 1 shows the eluted fraction of the Q-column, lane 2 represents the unbound flow through from the SP-column, lanes 3- 9 show the linear gradient of eluted fractions (5%, 10%, 20%, 30%, 40%, 80% and 100% respectively) and M represents the PageRuler Prestained Protein Ladder (ThermoFisher, Waltham, MA).

Table S1. O_2 affinities of recombinant nightjar Hb mutants.

Table S2. Museum-vouchered nightjar specimens used in the analysis of Hb structure and function.

Fig. S1

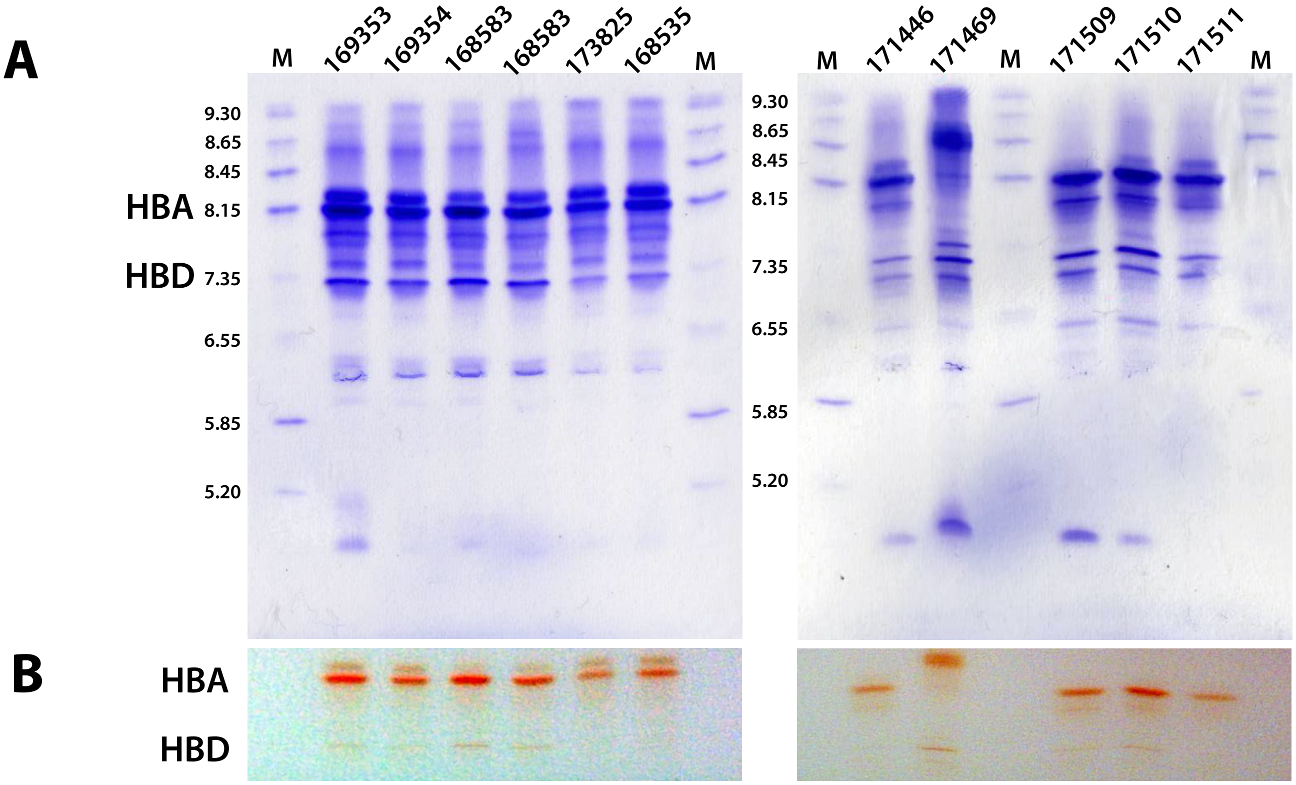
A
M
169353
169354
168583
168583
173825
168535
M
9.30
8.65
8.45
HBA
8.15
HBD
7.35
6.55
5.85
5.20
M
171446
171469
M
171509
171510
171511
M
9.30
8.65
8.45
8.15
7.35
6.55
5.85
5.20
B
HBA
HBD

Fig. S2

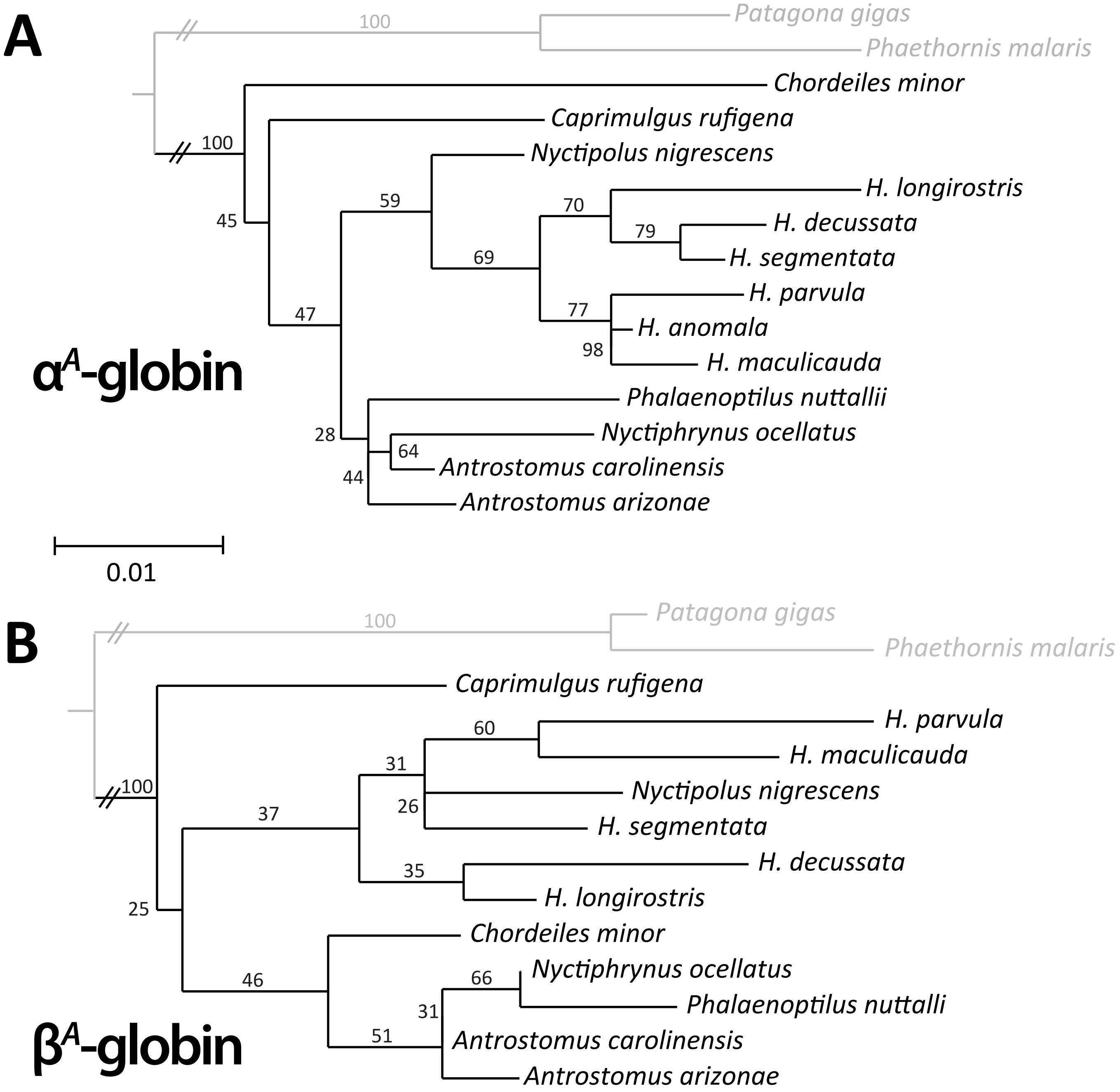
A
100
Patagona gigas
Phaethornis malaris
Chordeiles minor
Caprimulgus rufigena
100
Nyctipolus nigrescens
H. longirostris
59
70
H. decussata
45
79
69
H. segmentata
H. parvula
47
77
H. anomala
98
H. maculicauda
αA-globin
Phalaenoptilus nuttallii
Nyctiphrynus ocellatus
28
64
Antrostomus carolinensis
44
Antrostomus arizonae
0.01
B
100
Patagona gigas
Phaethornis malaris
Caprimulgus rufigena
60
H. parvula
H. maculicauda
31
Nyctipolus nigrescens
100
37
26
H. segmentata
35
H. decussata
25
H. longirostris
Chordeiles minor
66
Nyctiphrynus ocellatus
46
Phalaenoptilus nuttalli
31
51
Antrostomus carolinensis
βA-globin
Antrostomus arizonae

Fig. S3

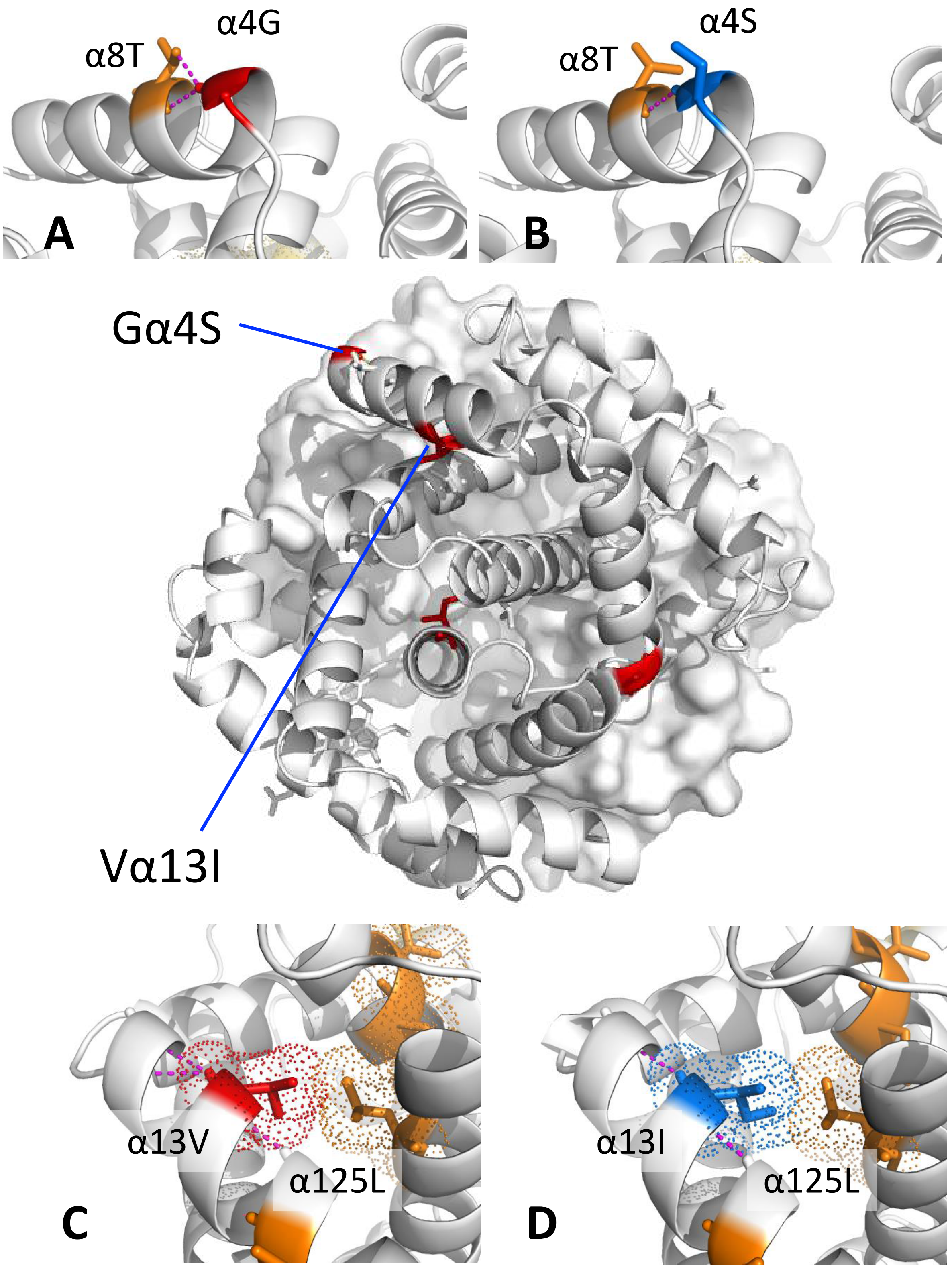

Fig. S4

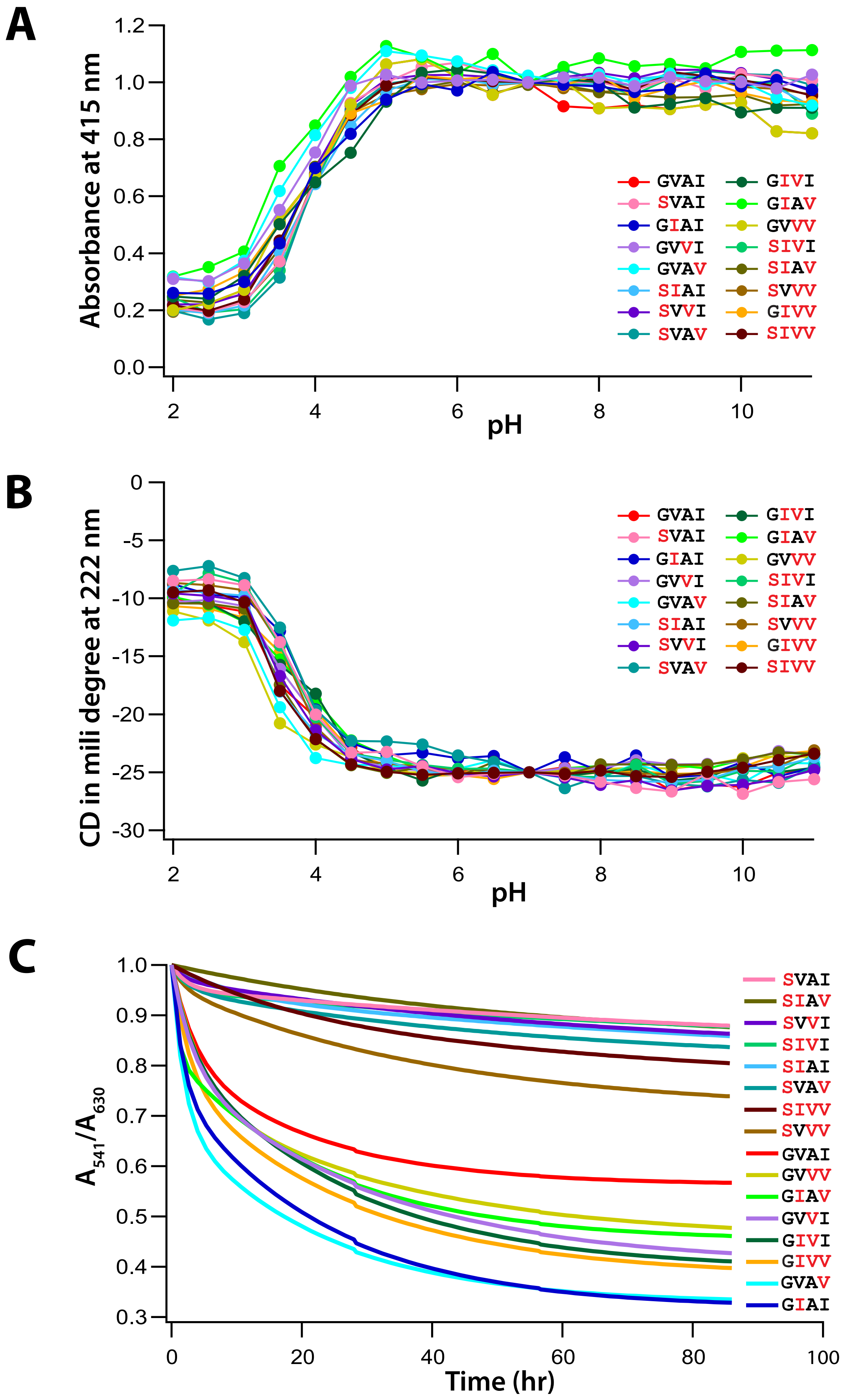
A
Absorbance at 415 nm
pH
GVAI
SVAI
GIAI
GVVI
GVAV
SIAI
SVVI
SVAV
GIVI
GIAV
GVVV
SIVI
SIAV
SVVV
GIVV
SIVV
B
CD in mili degree at 222 nm
pH
C
A541/A630
Time (hr)

Fig. S5

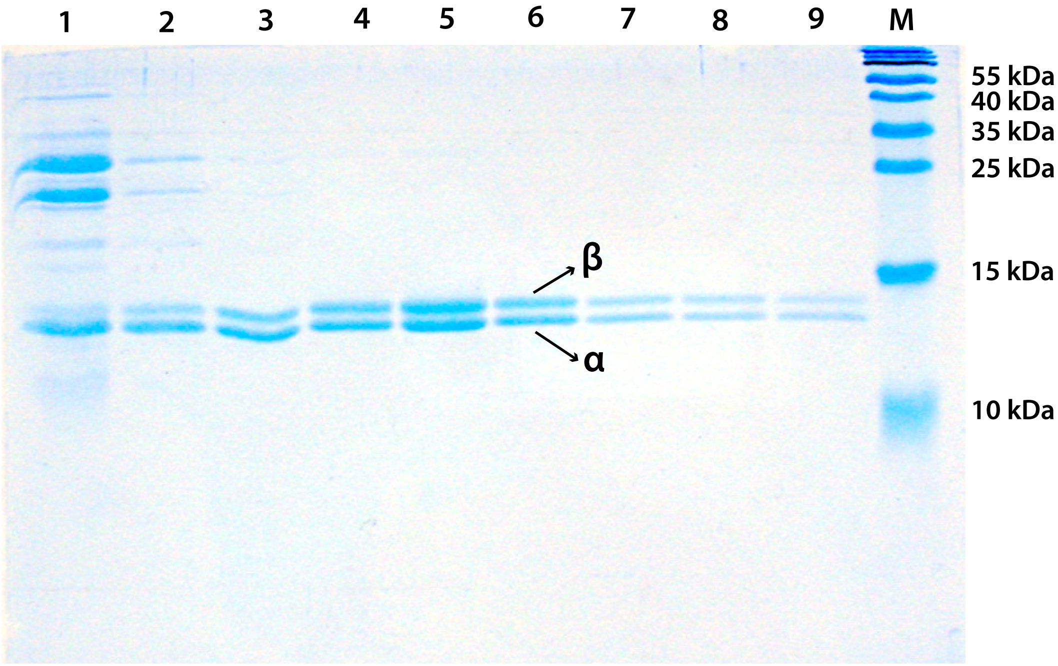

Table S1. O_2 affinities (P_{50}, torr; ± SE) of purified rHbs measured in 0.1 M HEPES buffer at pH 7.40, 37 °C. Measurements were conducted in the absence of anionic effectors (stripped) and in the presence of 0.1 M KCl and IHP (IHP/Hb tetramer ratio = 2.0). [Heme], 0.2 mM. Sensitivities to allosteric effectors were measured as the difference in log-transformed P_{50} values measured in the presence and absence of each effector, individually or in combination.

	GVAI	SVAI	GIAI	GVVI	GVAV	SIAI	SVVI	SVAV
P_{50} (torr)								
stripped	3.43±0.10	2.57±0.05	3.46±0.02	3.62±0.19	3.53±0.05	2.88±0.03	2.75±0.01	4.17±0.38
KCl+IHP	11.82±0.24	16.08±0.49	15.05±0.38	11.40±0.84	13.45±0.89	7.96±0.38	17.15±0.62	8.90±0.20
$\Delta\log P_{50([KCl+IHP]) - stripped)}$	0.54	0.80	0.64	0.50	0.58	0.44	0.80	0.33

	GIVI	GIAV	GVVV	SIVI	SIAV	SVVV	GIVV	SIVV
P_{50} (torr)								
stripped	2.90±0.05	3.74±0.24	3.81±0.13	3.46±0.31	3.44±0.06	3.15±0.08	3.83±0.04	2.88±0.08
KCl+IHP	12.54±0.22	10.55±0.37	12.30±0.63	6.27±0.24	16.39±1.00	17.49±1.07	11.98±0.13	18.91±2.01
$\Delta\log P_{50([KCl+IHP]) - stripped)}$	0.64	0.45	0.51	0.26	0.68	0.74	0.50	0.82

Table S2. Museum-vouchered nightjar specimens used in the analysis of Hb structure and function. The URL associated with each individual specimen provides a link to complete data on the open-access Arctos database. Frozen tissue and voucher specimens with 'NK' prefixes are stored at the Museum of Southwestern Biology (New Mexico, USA) and CORBIDI (Lima, Peru), and those with 'KUNHM' prefixes are stored at the University of Kansas Natural History Museum.

Species	Elevation (m)	Specimen ID	URL with MSB Catalog Number
Hydropsalis longirostris	4401	NK169353	http://arctos.database.museum/guid/MSB:Bird:34127
Hydropsalis longirostris	4401	NK169354	http://arctos.database.museum/guid/MSB:Bird:34128
Hydropsalis longirostris	4384	NK169307	http://arctos.database.museum/guid/MSB:Bird:34081
Hydropsalis longirostris	3940	NK168583	http://arctos.database.museum/guid/MSB:Bird:33364
Hydropsalis longirostris	3931	NK173825	http://arctos.database.museum/guid/MSB:Bird:35994
Hydropsalis longirostris	3927	NK168535	http://arctos.database.museum/guid/MSB:Bird:33316
Hydropsalis longirostris	3300	NK162746	http://arctos.database.museum/guid/MSB:Bird:28187
Hydropsalis longirostris	3120	NK159748	http://arctos.database.museum/guid/MSB:Bird:27091
Hydropsalis decussata	309	NK171446	http://arctos.database.museum/guid/MSB:Bird:34720
Hydropsalis decussata	309	NK171469	http://arctos.database.museum/guid/MSB:Bird:34743
Hydropsalis decussata	309	NK171509	http://arctos.database.museum/guid/MSB:Bird:34783
Hydropsalis decussata	309	NK171510	http://arctos.database.museum/guid/MSB:Bird:34784
Hydropsalis decussata	309	NK171511	http://arctos.database.museum/guid/MSB:Bird:34785
Hydropsalis maculicauda		KUNHM5844	
Hydropsalis segmentata	2850	NK171342	http://arctos.database.museum/guid/MSB:Bird:34616
Hydropsalis anomala		KUNHM3275	
Hydropsalis parvula		KUNHM106	
Chordeiles minor	1700	NK35865	http://arctos.database.museum/guid/MSB:Bird:40330
Caprimulgus rufigena	1250	NK174134	http://arctos.database.museum/guid/MSB:Bird:30050
Antrostomus carolinensis	10	NK142201	http://arctos.database.museum/guid/MSB:Bird:25325
Antrostomus arizonae	1610	NK103268	http://arctos.database.museum/guid/MSB:Bird:23095
Nyctipolus nigrescens	1423	NK175119	http://arctos.database.museum/guid/MSB:Bird:36288
Phalaenoptilus nuttalli	1815	NK165384	http://arctos.database.museum/guid/MSB:Bird:26711
Nyctiphyrnus ocellatus	1675	NK175047	http://arctos.database.museum/guid/MSB:Bird:36216
